# Identification of conserved proteins from diverse shell matrix proteome in *Crassostrea gigas*: characterization of genetic bases regulating shell formation

**DOI:** 10.1038/srep45754

**Published:** 2017-04-04

**Authors:** Dandan Feng, Qi Li, Hong Yu, Lingfeng Kong, Shaojun Du

**Affiliations:** 1 Key Laboratory of Mariculture, Ministry of Education, Ocean University of China, Qingdao 266003, China; 2 Laboratory for Marine Fisheries Science and Food Production Processes, Qingdao National Laboratory for Marine Science and Technology, Qingdao 266071, China; 3 Institute of Marine and Environmental Technology, Department of Biochemistry and Molecular Biology, University of Maryland School of Medicine, Baltimore, MD, United States

## Abstract

The calcifying shell is an excellent model for studying biomineralization and evolution. However, the molecular mechanisms of shell formation are only beginning to be elucidated in Mollusca. It is known that shell matrix proteins (SMPs) play important roles in shell formation. With increasing data of shell matrix proteomes from various species, we carried out a BLASTp bioinformatics analysis using the shell matrix proteome from *Crassostrea gigas* against 443 SMPs from nine other species. The highly conserved tyrosinase and chitin related proteins were identified in bivalve. In addition, the relatively conserved proteins containing domains of carbonic anhydrase, Sushi, Von Willebrand factor type A, and chitin binding, were identified from all the ten species. Moreover, 25 genes encoding SMPs were annotated and characterized that are involved in CaCO_3_ crystallization and represent chitin related or ECM related proteins. Together, data from these analyses provide new knowledge underlying the molecular mechanism of shell formation in *C.gigas*, supporting a refined shell formation model including chitin and ECM-related proteins.

An amazing diversity of mineralized structures to fulfill a host of biological functions have been constructed by animal life since the Phanerozoic Eon^1^. The products of these biomineralization processes range from individual functional elements, for example, shell, teeth, bone, to large scale geological structures, such as coral reefs^2^. Ever since the onset of metazoan biomineralization at the dawn of the Phanerozoic, their biomineralized structures have been mostly constructed from one of three minerals: Calcium carbonate (CaCO_3_), calcium phosphate (CaPO_4_), or silica (SiO_2_)^3^. CaCO_3_ products include in most brachiopod shells, molluscan shells, coral skeletons, echinoderm spines and tests, and sponge spicules^2^. The calcifying shell primarily consists of aragonite and/or calcite polymorphs, and exhibits prismatic, nacreous, foliate, cross-lamellar or homogenous microstructures^4^.

The calcifying shell serves as an excellent model for studying the process of biomineral formation and its evolution^5^. However, the molecular mechanisms by which shells are constructed are only beginning to be elucidated^6^,^7^. It has been shown that in addition to the major component of CaCO_3_, the shell is an extracellular ensemble of organic macromolecules including proteins, pigments, glycoproteins, lipid and polysaccharides. These organic materials are secreted by an evolutionarily homologous organ known as the mantle^1^,^8^. The mantle is divided into distinct morphogenetic regions consisting of highly specialized epithelial cell types^9^. The mantle produces shell matrix proteins (SMPs) that play important roles in crystal nucleation, polymorphism, morphology, and organization of CaCO_3_ crystallites during shell formation^10^.

In order to study the phylogenetic relationships among molluscs and refine the existing biomineralization models, it is necessary to understand the repertoire involved in shell formation. Many studies have been focused on the SMPs and genes expressed in the mantle, leading to the identification and characterization of several specific shell proteins^11^. Recently, high-throughput transcriptomics and proteomics analyses have been successively used to characterize shell matrix proteomes (SMPEs) in *Haliotis asinina*^8^, *Pinctada margaritifera*^12^, *Pinctada maxima*^12^, *Crassostrea gigas*^13^, *Lottia gigantea*^14^, *Cepaea nemoralis*^6^, *Mytilus coruscus*^15^, *Pinctada. fucata*^10^, *Magellania venosa*^2^, *Mytilus galloprovincialis*^16^, *Mya truncata*^17^, and *C. gigas, Mytilus edulis*, and *Pecten maximus*^18^. However, only two global SMPEs comparisons have been performed in *C. nemoralis* and *M. venosa*, revealing low similarities. Even in bivalves, two different models have been proposed for calcifying shell formation. The well-known matrix model of “chitin-silk fibroin gel proteins-acidic macromolecules” largely explained biomineralization in *P. fucata* from a crystal growth perspective^10^,^19^. While the cellular model shows that shell formation is orchestrated by cells and the extracellular matrix (ECM) and crystals are formed in haemocytes in eastern oyster, *Crassostrea virginica*^20^,^21^.

As sessile marine animal living in estuarine and intertidal regions, oysters must cope with harsh and dynamically changing environments under the protection of hard shell^13^. The Pacific oyster, *C. gigas,* is a potential model organism for marine molluscan due to extensive studies of biology and genetics and its economical, biological and ecological importance^22^. Up to date, only two genes related to shell formation have been cloned and characterized in *C. gigas*^23^,^24^. A previous study of SMPE consisting of 259 SMPs (SMPE-Cg1) suggested that the matrix model may not apply to *C. gigas* because the absence of silk-like proteins^13^. Recently, a SMPE of *C. gigas* consisting of 53 SMPs (SMPE-Cg2) was published^18^, providing new opportunities for comprehensive comparison and characterization of SMPs in marine molluscan.

Here, we compared two published SMPEs in *C. gigas* and chose SMPE consisting of 53 SMPs for global comparison against 443 SMPs from nine other species. A total of 58 SMPs were used to identify the homologous SMPs in *C. gigas* and analyze the molecular mechanisms of shell formation. Data from this study will assist our understanding of shell biomineralization and evolution.

## Result and Discussion

### *C. gigas* SMPE characterized by ID

With the SMPE-Cg2 published by Arivalagan *et al*., the integrated dataset with 76 SMPs from this work (SMPE-Cg3, refer to material and methods) and the SMPE-Cg1 from Zhang *et al*., in hand, we performed a global SMP comparison analysis. Comparing gene accession of SMPs, 39 proteins were found in both the dataset with 53 SMPs and 259 SMPs of which 31 SMPs were found in the integrated dataset with 76 SMPs. By BLASTp research, 45 proteins were identified from both the SMPE-Cg1 and the SMPE-Cg2 to have similar domains, of which 41 were found in the SMPE-Cg3. SMPE-Cg3 included in the majority of SMPs stably identified from both the SMPE-Cg1 and the SMPE-Cg2, suggesting that the majority of SMPs stably identified from the SMPE-Cg1 and the SMPE-Cg2 are specially or highly expressed in mantle. Considering that SMPE-Cg2 has more stably identified SMPs than the SMPE-Cg3 and is constructed using the same method as the other nine SMPEs, we conducted the subsequent comparison analysis using SMPE-Cg2.

It has been shown that proteins containing the repeated low-complexity domain (RLCD) are a prominent feature of all shell-forming proteomes, and most of which are found to be lineage-specific proteins^7^,^14^. The RLCDs are important implications for intrinsically unstructured/disordered (ID) proteins. ID proteins possess low binding affinity for other organic macromolecules, but weakly bind mineral surfaces and ions in aqueous phases, which is critical for matrix assembly^14^,^25^. In *C. gigas* SMPEs, 66 out of the SMPE-Cg3 and 47 out of the SMPE-Cg2 were predicted to have at least one region of ID (Supplementary Table S1). This is higher than that found in *P. fucata* which contained 22 ID proteins out of the 35 SMPs^10^. XSTREAM analysis predicted that 24 out of SMPE-Cg3 and 21 out of SMPE-Cg2 proteins to contain tandem repeats in *C. gigas,* which is higher than that reported in *P. fucata (*7 out of 35). Collectively, the SMPE-Cg2 contained more ID proteins than the SMPE-Cg3. Given that the SMPE is dominated by ID proteins within a wide range of molluscan shells, it is proposed that they might play crucial roles in shell formation or imparting to the shell certain physical properties such as fraction resistance.

### Comparisons of shell matrix proteomes

Up to date, SMPEs of 13 species belonging to different taxons have been published and includes *H. asinina* 8*, P. margaritifera*^12^*, P. maxima*^12^*, C. gigas*^13^*, L. gigantea*^14^*, C. nemoralis*^6^*, M. coruscus*^15^*, P. fucata*^10^*, M. venosa*^2^*, M. galloprovincialis*^16^*, M. truncata*^17^*, and M. edulis, and P. maximus*^18^. In this work, SMPEs from nine species have been used to compare against that of *C. gigas*. SMPE in *M. galloprovincialis* was excluded for more than one EST mapped to each peptide. SMPEs in *P. maximus* and *M. edulis* were not published. These nine species belong to two phylums of Mollusca and Brachiopoda and two classes of Bivalve and Gastropod in Mollusca (Fig. S1). All ten datasets primarily consist of proteins isolated from the shells of the respective species, which are validated by transcriptomes or ESTs in shell-forming organs or genome databases.

**Figure 1:**
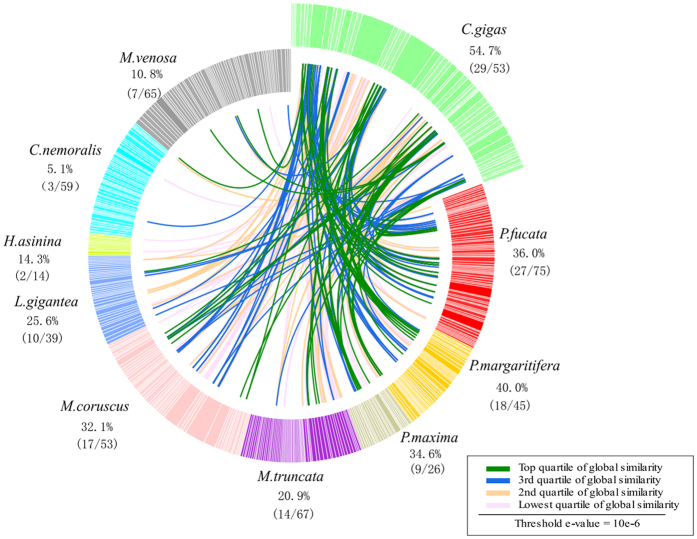
Comparisons of *C. gigas* shell proteome against nine other metazoan shell proteomes. BLASTp based similarity comparison of the 53 *C. gigas* SMPs against 443 shell-forming proteins derived from nine other biocalcifying metazoans, *P. fucata, P. margaritifera, P. maxima, M. truncata, M. coruscus, L. gigantea, H. asinina, C. nemoralis, M. venosa*. Individual lines spanning the ideogram connect proteins that share significant similarity (e-value < 10e-06). Transparent red lines connect proteins with the lowest quartile of similarity (with a threshold of 10 e-6) and green lines with the highest quartile of similarity. The percentage of each shell proteome that shared similarity with the *C. gigas* proteome is provided.

Our data showed that in *C. gigas*, 29 out of 53 (54.7%) SMPs shared sequence similarity (at an e-value threshold of 10e-06) with one or more proteins derived from other nine SMPEs investigated here (Fig. 1). Interestingly, SMPs in *C. nemoralis* showed a poor similarity of 5.1% (3 out of 59) with SMPs in *C. gigas*, compared to 10.8% (7 out of 65) in *M. venosa*. Obviously, the *C. gigas* in Mollusc shared a more recent common ancestry in evolutionary history with *C. nemoralis* than with *M. venosa* in Brachiopoda. However, *C. nemoralis* living on land is differentiated from other all nine species living in the sea by habitation. Marine and terrestrial shell-forming Mollusca have adapted to different environments that would fundamentally affect the process of shell formation and the stability of the secreted composite biomineral^6^. Therefore, the proteins and genetic bases involved in shell formation could be expected to have evolved rapidly in response to different selective pressures from different living environment.

Generally, the sequence similarity in Bivalve is greater than 30%, except *M. truncata* with a 20.9% similarity which was lower than *L.gigantea* with a 25.6% similarity in Gastropod. The mineralogy and shell microstructure of these species may account for that differences. The *C. gigas* shell is purely calcite: the outer shell layers are calcite prisms, the middle shell layers are chalky calcite and the inner shell layer are foliated calcite^18^. In contrast, *M. truncata* shell is composed only of aragonite. Other species in Mollusca are a combination of both calcitic and aragonitic layers. The imperfection of SMPE, speciation, and mineralogy might result in the relatively low similarity of 20.9–40.0% in Bivalve comparing with the 84.9% (45 SMPs in the SMPE-Cg2 have hits in the SMPE-Cg1 in *C. gigas*). It had been proposed that gastropod and bivalve nacre is the result of parallel evolution based on the large-scale differences in genes expression in nacre-forming cells of *Pinctada* and *Haliotis*^26^. Low and variable similarities of 5.1–25.6% in Gastropod imply the parallel evolution of shell between Gastropod and Bivalve. Collectively, comparison of the shell proteomes for sequence similarity gives some clues about the relationships between evolution and sequence conservation. However, it has been suggested that the similarity in SMPs depends not only on the evolutionary distance but is also influenced by mineralogy of shell, parallel evolution, adaptation to the environment etc^18^.

In order to identify the highly conserved SMPs, we ordered all 29 SMPs in *C.gigas* that were found in any other SMPEs according to the number of databases they were found in (Table S2). Four *C. gigas* SMPs were identified to have hits in all other five species in bivalve, which were annotated as Putative tyrosinase-like protein tyr-3 (Tyr-3), Histone-lysine N-methyltransferase MLL3 (MLL3), and Chitotriosidase-1. In molluscs, tyrosinase function has been implicated in seashell pigmentation and mineralization of seashell^27^,^28^. The tyrosinase family have gone through an expansion in bivalve. Chitotriosidase-1 consists of one region of glycohydro18_chitinase-like, which can hydrolyze chitin, the major non-protein component of mollusc shell^29^,^30^. The MLL3 proteins has a complex structure that consists of 16 domains. It was identified because of the tyrosinase region. These results emphasize the prominent role of tyrosinase and chitin related proteins in the shell formation of bivalve and many metazoan biominerals.

It should be noted that none of these proteins was found in all databases, suggesting that these SMPs are diverse or these databases are limited. Yet, ten proteins were found in at least five SMPEs, four of which are conserved genes in bivalve. Of that, Nacrein_like protein, whose homologue nacrein was the first shell matrix protein cloned from *P. fucata*, contains carbonic anhydrase (CA) domain. CA could control pH by converting CO_2_ to HCO_3_^−^ and concentrate CO_3_^2+^ to form amorphous CaCO_3_ in “chitin-silk fibroin gel proteins-acidic macromolecules” model^10^. Follistatin-related-protein was also identified, including chitin-binding 2 (CBD), immunoglobulin like (IG-like), and Lam-G. Lam-G is a Ca^+2^ mediated receptor, which has been implicated in cell adhesion and interaction. IG-like has been also implicated in cell adhesion and interaction. CBD can interact with chitin, which are usually found together with Von Willebrand factor type-A (VWA) in SMPs, like other three conservative proteins CGI_10009194, CGI_10028014, CGI_10012353. Both VWA and CBD are typically domains of collagen, the fundamental component of extracellular matrix (ECM). The identification of these conserved SMPs may help refine shell formation model, including the function of chitin and ECM related proteins in biomineralization framework and protein interactions.

### Identification and characterization of shell proteins homologues

Since the first SMP gene Nacrein was cloned, many SMPs have been cloned and characterized. Besides the shell’s superior mechanical and remarkable biocompatibility properties, the high commercial value of pearl has made pearl oyster one of the best studied biomineralization models. Here, we used these SMPs mainly from *P. fucata* to identify the homologues in *C. gigas* and analyzed the structural domains underlying biomineralization. A total of 58 SMP sequences were used to perform BLAST (Supplementary Table S3) and 25 protein sequences were annotated and characterized (Table 1). Furthermore, theses SMPs were classified into four categories based on functions and domains: crystallization of CaCO_3_, chitin related proteins, ECM related proteins and other proteins.

#### Crystallization of CaCO_3:_ ACCBP, CaLP, Nacrein

**Figure 2:**
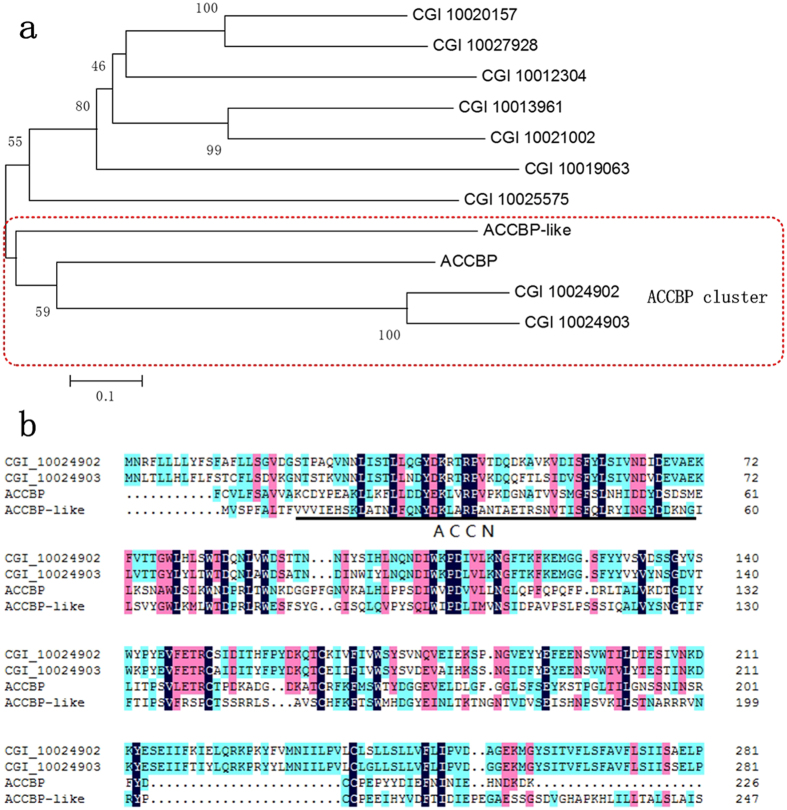
(**a**). Molecular phylogenetic tree of the nine ACCBP hits in *C. gigas* and two ACCBP in P. fucata. A phylogenetic tree was inferred from the amino acid sequences using the neighbor-joining method. Bootstrap values from 1000 trials are indicated at each branch node. The scale bar indicates 0.1 amino acid replacements per site. b. Comparison of neurotransmitter-gated ion-channel ligand binding domains (NCLBD) of ACCBP. The ACCN sequence is underlined.

Amorphous calcium carbonate-binding protein (ACCBP) is a member of the acetylcholine-binding protein family that was first isolated from the extrapallial fluid of *P. fucata*^31^. It has been reported that ACCBP inhibits undesired crystal growth and plays a key role in forming the exceedingly orderly microstructure of nacre^31^. ACCBP contains an ID region near the N-terminus (ACCN) that has been implicated in regulation of CaCO_3_ precipitation^32^. BLASTp search identified nine gene with sequence similarity to ACCBP (E-value < e-21, score > 100). Phylogenetic analysis of these nine genes formed two clades (Fig. 2), of which ACCBP, ACCBP-like, CGI_10024902, and CGI_10024903 were clustered into one clade. This suggests that CGI_10024902 and CGI_10024903 diverged from a common ancestral gene of ACCBP. These two genes are located on the same scaffold146, suggesting an intragenic duplication. Both proteins contain two neurotransmitter-gated ion-channel ligand-binding domains (NCLBD), as well as ACCBP and ACCBP-like. Sequence alignment showed that the conserved residues are different from those residues between ACCBP and the nAChR family (NCLBD-containing proteins in *D. melanogaster*)^32^, especially in the ACCN region (Fig. 2). There are significantly more conserved residues among ACCBP, ACCBP-like and the two proteins in ACCN sequence, which are assumed to play a role in mineralization activity.

Calmodulin-like protein (CaLP) is a multifunctional calcium sensor that belongs to a new member of the CaM superfamily^33^,^34^. In bivalve, CaLP contains two Ca^2+^-binding EF hand domains, each of which contains a pair of EF-hand motifs. Immunostaining revealed that CaLP was localized in the organic layer sandwiched between nacre (aragonite) and the prismatic layer (calcite) and the prismatic layer in *P. fucata*^35^. These results suggested that CaLP might be involved in the growth of nacre layer and prismatic layer. We have identified three putative genes encoding CaLP using BLASTn search (E-value < E-05, score > 50). BLASTp search identified 26 gene models (E-value < E-05 and score > 100), which included the aforementioned three gene models in the best six hits. Using the best six protein sequences in BLASTp, the phylogenetic tree showed that CGI_10011294 and CaLP constitute a single clade, as well as CGI_10011293 and CaM (Supplementary Fig S2). Therefore, we annotated CGI_10011294 as CaLP, and CGI_10011293 as CaM.

Nacrein is the first identified molluscan organic matrix component^36^. Nacrein expressed throughout the entire mantle epithelium and it functions in the production of both prismatic and nacreous layer^37^,^38^. Nacrein contains two functional domains, a CA and a NG-repeat domain with a repeated sequence rich in Asn and Gly^36^. Nacrein-related proteins have been found in some congeneric species (*P. maxima* and *P. margaritifera*) and also one gastropod, *Turbo marmoratus*^39^. They have a similar primary structure as nacrein in *P. fucata*^11^. No nacrein-related genes could be identified using BLASTp search with E-value < E-21 and score > 100. However, four nacrein-related genes were identified with E-value < E-05 and score > 80. Sequence alignment showed that the four hits have no NG-repeat domain and corresponding active site^39^. Phylogenetic tree of the aforementioned protein sequences showed two different clusters of nacrein group and hits group (Supplementary Fig. S3). These four hits are not specifically or highly expressed in mantle. Taken together, we argue that the four hits as CA, but not nacrein, suggesting the NG-repeat domain in CA proteins was acquired independently in the lineages of bivalves and gastropods.

These three SMPs have been reported to participate in concentration of Ca^2+^ and CO_3_^2−^, crystal nucleation, and inhibition of crystal growth.

#### Chitin Related Proteins: Chitin synthase, Clp

Chitin existing in both nacreous and prismatic organic matrix is thought to play important role in biomineralization^40^. Chitin synthases are expressed in mantle edge, contributing to the formation of the framework for shell calcification^29^. Chitin synthases characterized so far comprises three domains, A, B, and C^29^. It is noteworthy that the chitin synthase identified in Mollusca share a special feature-a myosin domain in the N-terminus^29^,^41^. We have identified two predicted gene models (CGI_10009438, CGI_10012656) that are homologous to chitin synthase using BLASTn search (E-value < E-63, score > 250). The two predicted gene models were located two different scaffolds. They encode two different predicted proteins that were also identified by BLASTp with E-value = 0, score > 1800.

**Figure 3:**
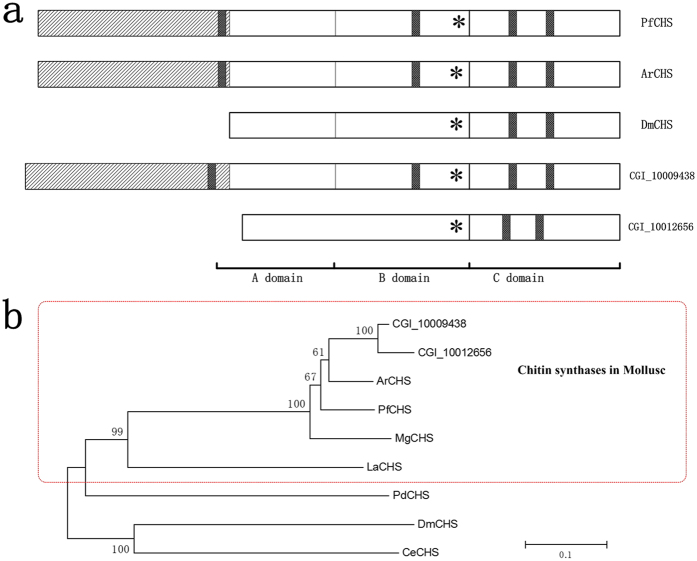
Diagrams of domain structures and phylogenetic tree analyses. (**a**). Diagrammatic representations of the domain structure of chitin synthase in different species. The myosin head domain is represented by a slashed box. Predicted coiled-coil regions are partially shaded. Asterisks represent the position of the chitin synthase active site (QRRRW), which is used to align the diagram. The three domains in these proteins are denoted A, B, and C. PfCHS, ArCHS and DmCHS are chitin synthases from *Pinctada fucata, Atrina rigida* and *Drosophila melanogaster*^66^. (**b**). A phylogenetic tree showing the evolutionary relationship of the predicted chitin synthases in *C. gigas* with known chitin synthases. The tree was constructed using maximum likelihood method. Sequences in the QRRRW catalytic domain were aligned to generate the tree. Numbers on the nodes indicate bootstrap values. ArCHS (AAY86556.1), PfCHS (BAF73720.1), MgCHS (ABQ08059.1), LaCHS (AHX26699.1), PdCHS (AHX26708.1), DmCHS (AAG09735.1) and CeCHS (NP_493682.2) are chitin synthases from *A. rigida, P. fucata, Mytilus galloprovincialis, Leptochiton asellus, Platynereis dumerilii, Drosophila melanogaster, Caenorhabditis elegans*.

We aligned the amino acid sequences of chitin synthases and discovered that these two proteins included all domains (a myosin domain and A, B, C domains) in *PfCHY* (Fig. 3). COILS analysis of CGI_10009438 showed a strong potential for coiled coil formation at four positions with *PfCHS*. Specifically, three positions showed a strong potential and one position showed a fairly weak potential. COILS analysis of CGI_10012656 showed strong potential for coiled coil formation at the same two relative positions with DmCHS. The phylogenetic tree strongly confirmed that the putative chitin synthase from *C.gigas* belongs to the chitin synthase family (Fig. 3). The mollusk chitin synthase group including PfCHS, ArCHS, MgCHS is separated from other chitin synthase groups by forming an independent cluster. This position of CgCHS in the phylogenetic tree is consistent with the evolutionary position in Mollusca. These data suggested that the chitin synthase of molluscan species gained the coiled-coil sequence in the N-terminal region during evolution.

Chitinase-like proteins (Clp) were first detected from *P. margaritifera* and *P. maxima*. Clp transcripts were localized in the mantle edge specifically implicated in the biomineralization of the prisms^12^. Clp3 in *P. margaritifera* includes in a region of GH18_chitinase-like, which can hydrolyze chitin. Using the Clp3 sequences, 14 gene models are identified by BLASTp with E-value < E-39, Score > 150. Of that, three hits CGI_10026599, CGI_10024867, CGI_10026605 are detected to specially express in mantle of *C.gigas*^13^, which strongly suggested that these gene models are related with shell formation. These three hits contain GH18 domain, showing hydrolysis of chitin activity.

Both of chitin synthase and chitinase play key roles in construction and reconstruction of chitin framework, just like “chitin-silk fibroin gel proteins-acidic macromolecules” model.

#### ECM related Proteins: Pif and BMSP, EGF-ZP, SPARC

Pif is an acidic matrix protein which regulates nacre formation. It was first identified in the pearl oyster *P. fucata.* The Pif gene encoded a precursor protein, which was posttranslationally cleaved to produce Pif 97 and Pif 80, respectively. Pif 97 has two conserved domains, a VWA domain for protein-protein interaction and chitin-binding domain. Pif 80 has aragonite-binding activity^42^. Sequence analysis revealed that blue mussel shell protein (BMSP) is a Pif homologous preproprotein in *M. galloprovincialis*. BMSP consisted of a signal peptide and two proteins, BMSP 120 and BMSP 100, respectively^43^. In addition, other Pif homologues from bivalves (*P. margaritifera, P. maxima*, and *Pteria penguin*) and gastropods (*L. gigantea*) have been identified, suggesting a common ancestral gene during evolution^43^.

**Figure 4:**
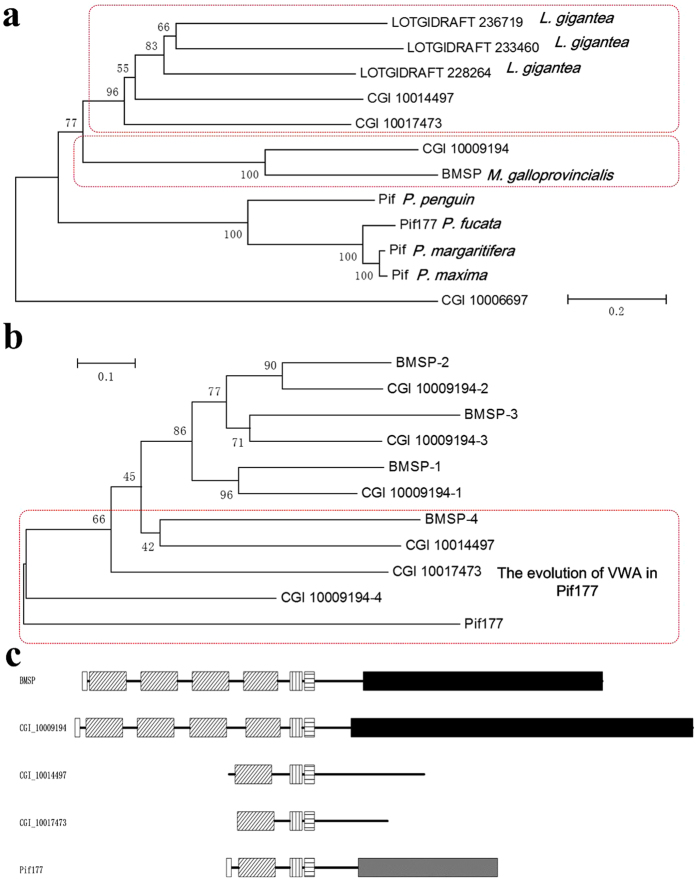
Phylogenetic tree of Pif and BMSP proteins in Mollusca. The tree was constructed using the NJ method, and the number at each node shows the bootstrap value.

We have identified six putative BMSP genes by BLASTp search (E-value < E-05 and score > 100), but no putative BMSP gene hits using BLASTn search (E-value < E-05, score > 50). Of that, the gene CGI_10009194 is homologous to BMSP with E-value = 0, score = 1415. Another two genes, CGI_10014497 and CGI_10017473, consist of the VWA domains and a CBD. We have identified ten putative Pif genes by BLASTp (E-value < E-05 and score > 100) using Pif177, but none using BLASTn search (E-value < E-05, score > 50). In addition to the former three genes (CGI_10014497, CGI_10017473, CGI_10009194), one more gene (CGI_10006697) with both VWA domains and a CBD is considered to be homologue of Pif, others have neither VWA domain no CBD. In phylogenetic analysis, the CGI 10009194 and BMSP formed a separate clade, suggesting that CGI 10009194 should be annotated as BMSP. The CGI 10014497 and CGI 10017473 clustered with Pif genes from *L. gigantea* with a low confidence value. CGI 10006697 formed a single clade with others and showed distant evolution, not being annotated as Pif (Fig. 4). Phylogenetic analysis of VWA domain showed that the former three VWA domains in CGI 10009194 formed a single clade consistently with the former three in BMSP that were evolved from BMSP-4 and CGI 10009194-4 (Fig. 4). These data indicated that BMSP and CGI 10009194 were likely evolved from an ancestral with four VWA domains. The phylogenetic tree showed that VWA domains in CGI 10014497 and CGI 10017473 have evolved from CGI 10009194-4 and Pif177, being annotated as *Pif-like1* and *Pif-like2.* Schematic representation of Pif showed the common VWA domains, CBDs, chitin-binding like domains and various C-terminal aragonite-binding sequences (Fig. 4).

Epidermal growth factor (EGF) domain-containing SMPs were first identified from *C. gigas*^44^ and named as Cgigas-IMSP-2.They were subsequently discovered in *P. maxima, P. margaritifera,* and *L. gigantea*^12^,^14^. Generally, the SMP consists of both EGF-like domain and one zona pellucida (ZP) domain. The presence of both domains in one protein is uncommon^12^. EGF-like domains, which are characterized by six conserved cysteines linked in a characteristic pattern of disulfide bonds and two small β-sheets, are among the smallest but most widely distributed modules found in extracellular proteins. EGF-like domains occur in a variety of proteins associated with diverse biological functions such as cell adhesion, signaling, and Ca^2+^-binding^45^. The ZP domains are present in a range of extracellular filament or matrix proteins. The ZP domain is characterized by eight conserved cysteine residues, which are involved in protein polymerization. The specific function of the EGF- and ZP-containing SMPs in calcified shell biomineralization await validation. Three EGF-ZP genes were identified by BLASTp using the known IMSP-2 genes of *C. gigas* with (E-value < E-48, score > 100). CGI_10017543, CGI_10017544, and CGI_10017545 are located on the same scaffold 120. In combination with their relatively high degree of sequence identify (37.53%), it strongly suggests that they originated from a gene duplication event. A sequence alignment of these EGF-ZPs illustrated a strong conservation of each domain (signal peptide, EGF, ZP), suggesting a fundamental role in biomineralization (Fig. S4).

SPARC (secreted protein, acidic, rich in cysteine), also known as BM-40 or osteonectin, is a major noncollagenous matrix protein of bone and a common mineralization-related proteins of vertebrates and molluscs^45^. The primary structure of SPARC is characterized by the presence of three functional domains: the N-terminal acidic domain I; the follistatin-like domain II with 10 conserved cysteine residues; and the C-terminal domain III, which is involved in interactions with collagen molecules^46^. By searching the *C. gigas* genome, we could only identify a single SPARC gene (CGI_10005088). Thus, the *C. gigas* genome contains one SPARC gene as observed in most triploblastic organisms. The SPARC protein consists of a region Kazal type serine protease inhibitors, follistatin-like domains (KAZAL_FS), and a region extracellular Ca^2+^ binding domain (SPARC_EC). KAZAL_FS can inhibit serine proteases and play an important role in tissue specific regulation. SPARC_EC functions to regulate cell-matrix interactions and binds to proteins such as collagen and vitronectin. The EC domain interacts with a follistatin-like (FS) domain which appears to stabilize Ca^2+^ binding.

These ECM related proteins are involved in crystallization, chitin-binding, and interactions with macromolecules. Typically, Pif is modularly constructed with aragonite-binding domain, chitin-binding domain, and protein-protein interaction domain, strongly suggesting that each shell protein is able to perform different functions. ECM-related domains in the SMPs are generally related with mediating communication between cells and the extracellular matrix.

#### Other Proteins of Interest: Tyrosinase, Peroxidase

Tyrosinases belong to the type-3 copper protein superfamily that also include tyrosinase-related proteins, arthropod phenoloxidases, and widespread hemocyanins. These protein possess a conserved pair of copper-binding domains^24^,^47^. Tyrosinases can be classified into three subclasses of secreted (α), cytosolic (β), and membrane-bound (γ) based on domain architecture and conserved residues in the copper-binding sites^48^. Tyrosinase is a key enzyme that mediates the hydroxylation of monophenols and oxidation of *o-*diphenols. It is thus also classified as a phenoloxidase. Tyrosinase is well known for its key biological role in melanin biosynthesis via transformation of tyrosine to L-DOPA. Tyrosinase functions in pigmentation and innate immunity^47^. In addition, other products of the melanin pathway participate in cuticle sclerotization in insects^49^.

In Mollusca, tyrosinase have been suggested in pigmentation and biomineralization of seashell. Cephalopod tyrosinases are expressed in the ink sac, suggesting an important role in melanin production^50^. In *P. fucata*, three tyrosinase genes have been characterized, Pfty1 and Pfty2 are suggested to function in prismatic formation and OT47 is proposed to influence the periostracum formation^51^,^52^. In *C. gigas, Cgtyr1* was cloned and proposed to specifically function in the initial phase of the larval shell biogenesis^23^. *CgTyr2* was also cloned and showed high levels of expression in mantle edges. It has been suggested to play a role in the formation of periostracum/pigmentation^24^. These reports strongly suggest that tyrosinases play diverse roles in stages when seashell is constructed, pigmented, and covered with the periostracum.

**Figure 5:**
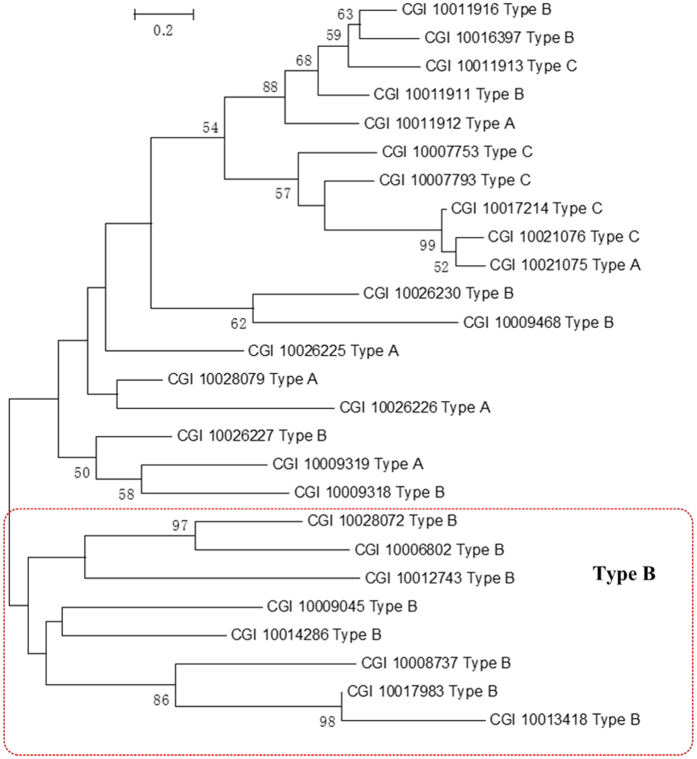
Tyrosinase genes diversity in *C. gigas*. The phylogenetic tree of tyrosinases was constructed by the maximum likelihood method. Numbers on the nodes indicate bootstrap values. The tyrosinase genes are named by accession number and type.

Twenty six tyrosinase genes were identified from *C. gigas* genome. Two tyrosinase genes CGI_10007793 and CGI_10011913 were found to be identical to the reported *Cgtyr1* and *CgTyr2,* respectively (Fig. 5). The tyrosinase gene family can be further classified into three types: secreted form with signal peptides (Type A), cytosolic form (Type B) and member-bound form (Type C). According to SignalP v4.0, and TMHMM Server v2.0, there are six TypeA tyrosinase genes, 15 Type B tyrosinase genes and five Type C tyrosinase genes. The phylogenetic tree of 26 tyrosinases showed that there are eight pairs of duplication genes. Among them, only two pairs of CGI_10021076 Type C and CGI_10021075 Type A, CGI_10009319 Type A and CGI_10009318 Type B are located in the same scaffold separately, belonging to intragenic duplication. The phylogenetic tree showed that the clusters of tyrosinase genes are influenced by not only position of genes, but also the type of genes. The phylogenetic tree consists of two major groups, one of which consists of eight Type B tyrosinase genes. In the other group, CGI_10011916 Type B, CGI_10011913 Type C, CGI_10011911 Type B and CGI_10011912 Type A are clustered, all of which are located in Scaffold 43702. Above all, it suggests that the tyrosinases have evolved through both intergenic duplication and intragenic duplication.

**Figure 6:**
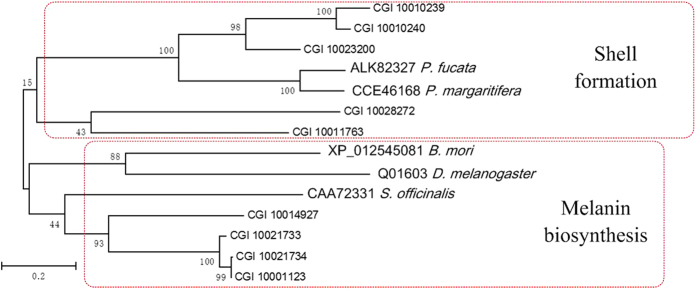
Peroxidase genes diversity in *C. gigas*. The phylogenetic tree of peroxidases was constructed by the maximum likelihood method. Numbers on the nodes indicate bootstrap values. The peroxidase genes are named by accession number and species.

Peroxidases are iron proteins that catalyse the oxidation of many aromatic amines and phenols by hydrogen peroxide. Inactivation experiments provide strong evidence that the DOPA reaction in the mantle of *Lymnaea stagnalis* is catalyzed by peroxidase, suggesting that peroxidase is involved in the quinone-tanning of periostracum proteins^53^. Peroxidase is exclusively expressed in the ink gland of *Sepia officinalis*, likely involved in melanin biosynthesis^54^. Typical peroxidase is characterized by a unique structural domain that contains two histidines (proximal and distal histidines) and one calcium-binding site, which was suggested to function in maintaining the protein structure in the heme environment^54^,^55^. Peroxidase has been retrieved from the shell matrix of *P. margaritifera* and *L. gigantea*. It was proposed to be involved in biomineral-hydrogel formation via protein matrix framework assembly^12^. We identified 26 peroxidase genes by BLASTp using the known peroxidase genes of *P. margaritifera* with (E-value < E-23, score > 100). The peroxidase genes have gone through an expansion as shown for tyrosinases in *C. gigas*. Nine out of the 26 peroxidase were specially or highly expressed in mantle of *C. gigas*. The two best hits (CGI_10023200 and CGI_10010240) are specifically expressed in mantle^13^. All nine peroxidases consist of two histidines and one calcium-binding site. Phylogenetic analysis showed that these peroxidases formed two clusters that could be identified as melanin biosynthesis group and the shell formation group (Fig. 6). Peroxidases from *Drosophila melanogaster, Bombyx mori* and *Sepia officinalis* have been implicated in melanin synthesis form melanin polymer^54^.

We noted in our bioinformatics analyses that in many cases, BLASTn gave no hits, however, BLASTp give hits (Table S2). This suggested that genes encoding SMPs have undergone more variation than SMPs protein sequences. Until now, literature on biomineralization is scattered with these identified SMPs. Obviously, SMPs do more than providing a framework for crystallization. They could be involved in interactions with other macromolecular components of the matrix and cells, giving subtle feedbacks between the shell and the calcifying mantle epithelium.

## Materials and Method

### Construction and characterization of SMPE in *C. gigas*

In *C. gigas*, a SMPE consisting of 53 proteins was recently published^18^. The SMPE was constructed from peptide fragments in shell, which can be mapped to the genome. In addition, a SMPE in *C. gigas* consisting of 259 proteins was also analyzed, which was constructed by Zhang and colleagues using the same method. Many house-keeping proteins, such as elongation factor 1α and ribosomal proteins, were found in these 259 SMPs, which are significantly more than SMPs identified from other molluscan shell proteome to date. Generally house-keeping proteins should not be found in shell matrix proteome and they do not play special role in biomineralization. Given that housekeeping genes are generally expressed at relatively constant levels in most non-pathological situations, we identified genes that are specifically or highly expressed in mantle to minimize the interference from housekeeping proteins. An integrated SMPE consisting of 76 SMPs was constructed. The revised SMPE was constructed from the intersection of two databases:1) 259 proteins isolated from oyster shells; 2) 492 genes that were specifically or highly expressed in mantle, of which, the highly expressed genes were defined as having RPFM values of at least 5 and at least five times of other organ average^13^. Intrinsically unstructured/disordered (ID) domains were predicted by IUPRED^56^ and XSTREAM^57^. IUPRED was used to recognize disordered regions from the amino acid sequences of SMPs based on the estimated pairwise energy content. XSTREAM was used to detect proteins with tandem-arranged repeat units in the default settings.

### Comparisons of shell-forming proteomes

Based on BLASTp, the global similarity comparison of *C. gigas* SMPE was performed against 443 SMPs derived from nine other biocalcifying metazoans. These included 53 *M. coruscus* proteins^15^, 75 *P. fucata* proteins^10^, 45 *P. margaritifera* proteins^12^, 26 *P. maxima* proteins^12^, 39 *L. gigantea* proteins^14^, 14 *H. asinina* proteins^8^, 59 *C. nemoralis* proteins^6^, 67 *M. truncata*^17^, and 65 *M. venosa*^2^ proteins. All protein sequences are validated by mapping to the transcriptomes, ESTs in biocalcifying organs or genome assemblies. The e-value threshold was set to 1e-06. These comparisons were made using blast+ 58: blastp -query XX.fa -db XX.change.fasta -out XX.blp -outfmt 6 -evalue 10e-6 -num_threads 10. The *.blp files generated by blast+ were modified using custom Perl scripts and then passed to Circos in order to generate an ideogram^59^. The *.blp file with details of the BLASTp results is provided (Supplementary Table S4).

### Identification of validated SMPs from *C. gigas* draft genome

The SMP searches in the *C. gigas* gene models (oyster_gene_v1) and induced protein sequences (oyster_peptides_v1) were performed using Oysterbase^13^. Shell formation complementary DNAs (cDNAs) in *C.gigas* were BLASTn and BLASTp searched. Identifies of the hit gene models associated with the original cDNAs were confirmed by sequence alignment. Similarly, SMPs identified from other molluscan species were BLAST searched, and the obtained gene models were reciprocally BLASTp searched against the NCBI nonredundant (nr) database to confirm the best-hit sequence.

### Characterization of homologous SMPs in *C.gigas*

The conserved structural domains were examined using the SMART^60^ and InterproScan^61^. The amino acid sequences were aligned using MEGA5^62^ or DNAMAN (Lynon Biosoft). For phylogenetic analysis, poorly aligned positions were checked and removed manually, the phylogenetic tree with bootstrap value was constructed using MEGA5. Protein sequences for the phylogenetic analysis were retrieved from GenBank, Swiss-Prot, or the Oysterbase. In the case of secretory proteins or peptides, the presence of a signal peptide was predicted by SignalP 4.0^63^. COILS was used to detect coiled coil formation^64^. To identify membrane proteins, the TMHMM Server v2.0 prediction algorithm was used for transmembrane helices^65^.

## Conclusion

We compared two different SMPEs of *C. gigas* consisting of 76 SMPs and 53 SMPs and chose the latter one to perform a broad level comparison by bioinformatic analysis. These SMPE were characterized by having a high proportion of ID proteins, especially RLCD proteins. We used a marine SMPE in *C. gigas* to perform a broad comparison against 443 SMPs from nine other species using BLASTp. Our data confirms the earlier findings that the SMPs similarity depends not only on the evolutionary distance but is influenced by mineralogy of shell, parallel evolution, adaptation to the environment etc. The highly conserved proteins tyrosinase and chitotriosidase are identified in bivalve, and the relatively conserved proteins with domains of CA, VWA, CBD, IG-like and LaG are identified from all ten species. 25 genes encoding SMPs were annotated and characterized that are chitin related or ECM related proteins involved in crystallization of CaCO_3_. These conserved SMPs and universal domains enrich the molecular knowledge of shell formation mechanism in *C. gigas*, urging for a refined shell formation model including both chitin and ECM-related proteins.

## Additional Information

**How to cite this article**: Feng, D. *et al*. Identification of conserved proteins from diverse shell matrix proteome in *Crassostrea gigas:* characterization of genetic bases regulating shell formation. *Sci. Rep.* **7**, 45754; doi: 10.1038/srep45754 (2017).

**Publisher's note:** Springer Nature remains neutral with regard to jurisdictional claims in published maps and institutional affiliations.

## Supplementary Material

Supplementary Information

## Figures and Tables

**Table 1:** Identification and characterization of SMPs from *C. gigas*.

Gene name	Categories	Expression in mantle^a^	Best matched gene ID	BLAST best hit to NCBI accession (species)	Shell layer^b^	Domains^c^
ACCBP1	CaCO3	N	CGI_10024902	EKC41060 (*C. gigas*)	—	NCLBD
ACCBP2	CaCO3	N	CGI_10024903	EKC41058 (*C. gigas*)	—	NCLBD
BMSP	Chitin	N	CGI_10009194	BAK86420 (*M. galloprovincialis*)	P,N	VWA;CBD
CaLP	CaCO3	N	CGI_10011294	P41041 (*Pneumocystis carinii*)	P,N	CaBEF
CaM	CaCO3	N	CGI_10011293	EKC20234 (*C. gigas*)	P,N	CaBEF
Cgtyr1	Others	S	CGI_10007793	EKC29813 (*C. gigas*)	—	CBS
CgTyr2	Others	S	CGI_10011913	EKC18549 (C. gigas)	p	CBS
Chitin synthase1	Chitin	H	CGI_10009438	AAY86556 (*Atrina rigida*)	P,N	MHD
Chitin synthase2	Chitin	N	CGI_10012656	BAF73720 (*P. fucata*)	P,N	MHD
chitobiase	Chitin	S	CGI_10007857	H2A0L6 (*P. margaritifera*)	P,N	Glyco_20
Chitotriosidase1	Chitin	S	CGI_10024867	AFO53261 (*Hyriopsis cumingii*)	P,N	Glyco_20
Chitotriosidase2	Chitin	S	CGI_10026605	CAI96027 (*C. gigas*)	P,N	Glyco_20
Clp3	Chitin	S	CGI_10026599	H2A0L5 (*P. margaritifera*)	P	Glyco_18
CopAmOx	ECM	H	CGI_10026457	EKC31553 (*C. gigas*)	P	Copper amine oxidase domain
EGF-ZP1	ECM	S	CGI_10017543	P86785 (*C. gigas*)	—	EGF;ZP
EGF-ZP2	ECM	S	CGI_10017544	EKC41439 (*C. gigas*)	—	EGF;ZP
EGF-ZP3	ECM	H	CGI_10017545	P86954 (*C. gigas*)	—	EGF;ZP
Fibronectin1	ECM	H	CGI_10016964	EKC41462 (*C. gigas*)	P	fibronectin type III
Fibronectin2	ECM	H	CGI_10016965	EKC41461 (*C. gigas*)	P	fibronectin type III
Peroxidase1	Others	S	CGI_10023200	EKC34657 (*C. gigas*)	—	CaBS
Peroxidase2	Others	S	CGI_10010240	EKC26108 (*C. gigas*)	—	CaBS
PFMG9	Others	H	CGI_10010153	ADC52432 (*P. fucata*)	—	KAZAL_FS
Pif-like1	Chitin	N	CGI_10014497	AKV63183 (*P. fucata*)	P,N	VWA;CBD
Pif-like2	Chitin	N	CGI_10017473	BAK86420 (*M. galloprovincialis*)	P,N	VWA;CBD
SPARC	ECM	N	CGI_10005088	AND99565 (*P. fucata*)		N-terminal acidic domain

^a^represents the gene expression levels in mantle compared with other organs: S = special, H = high, N = not special or high.

^b^represents the shell layer that were detected to contain the consistent proteins: p = periotracum, P = prismatic layer, N = nacreous layer; ‘P, N’ means that the SMPs were found in both the layers; “-” represents unknown.

^c^Abbreviation: CBS = copper-binding sites; MHD = myosin head domain; VWA = von Willebrand factor (vWF) type A domain; CBD = chitin-binding domain; CaBS = calcium-binding site; EGF = Epidermal growth factor; ZP = zona pellucida; CaBEF = Ca2+ -binding EF hand domain; NCLBD = neurotransmitter-gated ion-channel ligand-binding domains; KAZAL_FS = Kazal type serine protease inhibitors and follistatin-like domains; Glyco_18 = family 18 Glycosyl hydrolases; Glyco_20 = family 20 Glycosyl hydrolases.

## References

[b1] Simkiss K. & Wilbur K. M. Biomineralization(Elsevier, 2012).

[b2] Jackson D. J. . The *Magellania venosa* biomineralizing proteome: a window into brachiopod shell evolution. Genome biology and evolution 7, 1349–1362 (2015). 25912046 10.1093/gbe/evv074 PMC4453069

[b3] Knoll A. H. Biomineralization and evolutionary history. Reviews in mineralogy and geochemistry 54, 329–356 (2003).

[b4] M.Kocot K., Aguilera F., McDougall C., J.Jackson, & M.Degnan B. Sea shell diversity and rapidly evolving secretomes: insights into the evolution of biomineralization. Frontiers in Zoology 13, 1 (2016). 27279892 10.1186/s12983-016-0155-z PMC4897951

[b5] Marie B., Le Roy N., Zanella-Cleon I., Becchi M. & Marin F. Molecular evolution of mollusc shell proteins: insights from proteomic analysis of the edible mussel *Mytilus*. J Mol Evol 72, 531–46 (2011). 21643827 10.1007/s00239-011-9451-6

[b6] Mann K. & Jackson D. J. Characterization of the pigmented shell-forming proteome of the common grove snail *Cepaea nemoralis*. BMC genomics 15, 1 (2014). 24684722 10.1186/1471-2164-15-249 PMC4023409

[b7] Aguilera F., McDougall C. & Degnan B. M. Co-option and de novo gene evolution underlie molluscan shell diversity. Molecular Biology and Evolutiondoi, https://doi.org/10.1093/molbev/msw294 (2016). 10.1093/molbev/msw294 PMC5400390 28053006

[b8] Marie B. . Proteomic analysis of the organic matrix of the abalone *Haliotis asinina* calcified shell. Proteome Sci 8, 54 (2010). 21050442 10.1186/1477-5956-8-54 PMC2989941

[b9] Marin F. & Luquet G. Molluscan shell protein. Comptes Rendus Palevol 3, 469–492 (2004).

[b10] Liu C. . In-depth proteomic analysis of shell matrix proteins of *Pinctada fucata*. Scientific reports 5 (2015). 10.1038/srep17269 PMC4660305 26608573

[b11] Miyamoto H. . The diversity of shell matrix proteins: Genome-wide investigation of the pearl oyster, Pinctada fucata. Zoological science 30, 801–816 (2013). 24125645 10.2108/zsj.30.801

[b12] Marie B. . Different secretory repertoires control the biomineralization processes of prism and nacre deposition of the pearl oyster shell. Proceedings of the National Academy of Sciences 109, 20986–20991 (2012). 10.1073/pnas.1210552109 PMC3529032 23213212

[b13] Zhang G. . The oyster genome reveals stress adaptation and complexity of shell formation. Nature 490, 49–54 (2012). 22992520 10.1038/nature11413

[b14] Marie B. . The shell‐forming proteome of *Lottia gigantea* reveals both deep conservations and lineage‐specific novelties. FEBS Journal 280, 214–232 (2013). 23145877 10.1111/febs.12062

[b15] Liao Z. . In-depth proteomic analysis of nacre, prism, and myostracum of *Mytilus* shell. Journal of proteomics 122, 26–40 (2015). 25857279 10.1016/j.jprot.2015.03.027

[b16] Gao P. . Layer-by-Layer Proteomic Analysis of *Mytilus galloprovincialis* Shell. PLOS ONE 10 (2015). 10.1371/journal.pone.0133913 PMC4517812 26218932

[b17] Arivalagan J. . Shell matrix proteins of the clam, *Mya truncata*: Roles beyond shell formation through proteomic study. Marine Genomics 27, 69–74 (2016). 27068305 10.1016/j.margen.2016.03.005

[b18] Arivalagan J. . Insights from the shell proteome: biomineralization to adaptation. Molecular Biology and Evolution 34, 66–77 (2016). 27744410 10.1093/molbev/msw219 PMC5854119

[b19] Furuhashi T., Schwarzinger C., Miksik I., Smrz M. & Beran A. Molluscan shell evolution with review of shell calcification hypothesis. Comparative biochemistry and physiology Part B: Biochemistry and molecular biology 154, 351–371 (2009). 10.1016/j.cbpb.2009.07.011 19665573

[b20] Johnstone M. . Cellular orchestrated biomineralization of crystalline composites on implant surfaces by the eastern oyster, *Crassostrea virginica* (Gmelin, 1791). Journal of Experimental Marine Biology and Ecology 463, 8–16 (2015).

[b21] Mount A. S., Wheeler A. P., Paradkar R. P. & Snider D. Hemocyte-mediated shell mineralization in the eastern oyster. Science 304, 297 (2004). 15073378 10.1126/science.1090506

[b22] Yu H., Zhao X. & Li Q. Genome-wide identification and characterization of long intergenic noncoding RNAs and their potential association with larval development in the Pacific oyster. Scientific reports 6 (2016). 10.1038/srep20796 PMC4748301 26861843

[b23] Huan P., Liu G., Wang H. & Liu B. Identification of a tyrosinase gene potentially involved in early larval shell biogenesis of the Pacific oyster *Crassostrea gigas*. Development genes and evolution 223, 389–394 (2013). 23897397 10.1007/s00427-013-0450-z

[b24] Yu X. . Molecular cloning and differential expression in tissues of a tyrosinase gene in the Pacific oyster *Crassostrea gigas*. Molecular biology reports 41, 5403–5411 (2014). 24859978 10.1007/s11033-014-3412-2

[b25] Evans J. S. Aragonite-associated biomineralization proteins are disordered and contain interactive motifs. Bioinformatics 28, 3182–3185 (2012). 23060620 10.1093/bioinformatics/bts604

[b26] Jackson D. . Parallel evolution of nacre building gene sets in molluscs. Molecular biology and evolution 27, 591–608 (2010). 19915030 10.1093/molbev/msp278

[b27] Feng D., Li Q., Yu H., Zhao X. & Kong L. Comparative Transcriptome Analysis of the Pacific Oyster *Crassostrea gigas* Characterized by Shell Colors: Identification of Genetic Bases Potentially Involved in Pigmentation. PloS one 10 (2015). 10.1371/journal.pone.0145257 PMC4691203 26693729

[b28] Aguilera F., McDougall C. & Degnan B. M. Evolution of the tyrosinase gene family in bivalve molluscs: independent expansion of the mantle gene repertoire. Acta biomaterialia 10, 3855–3865 (2014). 24704693 10.1016/j.actbio.2014.03.031

[b29] Suzuki M., Sakuda S. & Nagasawa H. Identification of chitin in the prismatic layer of the shell and a chitin synthase gene from the Japanese pearl oyster, *Pinctada fucata*. Bioscience, biotechnology, and biochemistry 71, 1735–1744 (2007). 10.1271/bbb.70140 17617722

[b30] Weiss I. M., Schönitzer V., Eichner N. & Sumper M. The chitin synthase involved in marine bivalve mollusk shell formation contains a myosin domain. FEBS letters 580, 1846–1852 (2006). 16513115 10.1016/j.febslet.2006.02.044

[b31] Ma Z. . A novel extrapallial fluid protein controls the morphology of nacre lamellae in the pearl oyster, *Pinctada fucata*. Journal of Biological Chemistry 282, 23253–23263 (2007). 17558025 10.1074/jbc.M700001200

[b32] Amos F. F., Ndao M. & Evans J. S. Evidence of mineralization activity and supramolecular assembly by the N-terminal sequence of ACCBP, a biomineralization protein that is homologous to the acetylcholine binding protein family. Biomacromolecules 10, 3298–3305 (2009). 19904951 10.1021/bm900893f

[b33] Li S. . Cloning and expression of a pivotal calcium metabolism regulator: calmodulin involved in shell formation from pearl oyster (*Pinctada fucata*). Comparative Biochemistry and Physiology Part B: Biochemistry and Molecular Biology 138, 235–243 (2004). 10.1016/j.cbpc.2004.03.012 15253872

[b34] Li S., Xie L., Ma Z. & Zhang R. cDNA cloning and characterization of a novel calmodulin‐like protein from pearl oyster *Pinctada fucata*. Febs Journal 272, 4899–4910 (2005). 16176264 10.1111/j.1742-4658.2005.04899.x

[b35] Yan Z. . Biomineralization: functions of calmodulin-like protein in the shell formation of pearl oyster. Biochimica et Biophysica Acta (BBA)-General Subjects 1770, 1338–1344 (2007). 17692465 10.1016/j.bbagen.2007.06.018

[b36] Miyamoto H. . A carbonic anhydrase from the nacreous layer in oyster pearls. Proceedings of the National Academy of Sciences 93, 9657–9660 (1996). 10.1073/pnas.93.18.9657 PMC38484 8790386

[b37] Miyamoto H., Miyoshi F. & Kohno J. The carbonic anhydrase domain protein nacrein is expressed in the epithelial cells of the mantle and acts as a negative regulator in calcification in the mollusc *Pinctada fucata*. Zoological science 22, 311–315 (2005). 15795493 10.2108/zsj.22.311

[b38] Takeuchi T. & Endo K. Biphasic and dually coordinated expression of the genes encoding major shell matrix proteins in the pearl oyster *Pinctada fucata*. Marine biotechnology 8, 52–61 (2006). 16283581 10.1007/s10126-005-5037-x

[b39] Norizuki M. & Samata T. Distribution and function of the nacrein-related proteins inferred from structural analysis. Marine Biotechnology 10, 234–241 (2008). 18080162 10.1007/s10126-007-9061-x

[b40] Furuhashi T. . Pyrolysis GC/MS and IR spectroscopy in chitin analysis of molluscan shells. Bioscience, biotechnology, and biochemistry 73, 93–103 (2009). 10.1271/bbb.80498 19129649

[b41] Weiss I. M. & Schönitzer V. The distribution of chitin in larval shells of the bivalve mollusk *Mytilus galloprovincialis*. Journal of structural biology 153, 264–277 (2006). 16406681 10.1016/j.jsb.2005.11.006

[b42] Suzuki M. . An acidic matrix protein, Pif, is a key macromolecule for nacre formation. Science 325, 1388–1390 (2009). 19679771 10.1126/science.1173793

[b43] Suzuki M. . Identification and Characterisation of a Calcium Carbonate‐Binding Protein, Blue Mussel Shell Protein (BMSP), from the Nacreous Layer. Chembiochem 12, 2478–2487 (2011). 21932217 10.1002/cbic.201100317

[b44] Marie B., Zanella-Cléon I., Guichard N., Becchi M. & Marin F. Novel proteins from the calcifying shell matrix of the Pacific oyster *Crassostrea gigas*. Marine biotechnology 13, 1159–1168 (2011). 21537946 10.1007/s10126-011-9379-2

[b45] Maurer P. & Hohenester E. Structural and functional aspects of calcium binding in extracellular matrix proteins. Matrix biology 15, 569–580 (1997). 9138289 10.1016/s0945-053x(97)90033-0

[b46] Sasaki T., Hohenester E., Göhring W. & Timpl R. Crystal structure and mapping by site‐directed mutagenesis of the collagen‐binding epitope of an activated form of BM‐40/SPARC/osteonectin. The EMBO Journal 17, 1625–1634 (1998). 9501084 10.1093/emboj/17.6.1625 PMC1170510

[b47] García‐Borrón J. C. & Solano F. Molecular Anatomy of Tyrosinase and its Related Proteins: Beyond the Histidine‐Bound Metal Catalytic Center. Pigment Cell Research 15, 162–173 (2002). 12028580 10.1034/j.1600-0749.2002.02012.x

[b48] Aguilera F., McDougall C. & Degnan B. M. Origin, evolution and classification of type-3 copper proteins: lineage-specific gene expansions and losses across the Metazoa. BMC evolutionary biology 13, 96 (2013). 23634722 10.1186/1471-2148-13-96 PMC3658974

[b49] Andersen S. O. Insect cuticular sclerotization: a review. Insect biochemistry and molecular biology 40, 166–178 (2010). 19932179 10.1016/j.ibmb.2009.10.007

[b50] Naraoka T. . Purification, characterization and molecular cloning of tyrosinase from the cephalopod mollusk, *Illex argentinus*. European Journal of Biochemistry 270, 4026–4038 (2003). 14511385 10.1046/j.1432-1033.2003.03795.x

[b51] Nagai K., Yano M., Morimoto K. & Miyamoto H. Tyrosinase localization in mollusc shells. Comparative Biochemistry and Physiology Part B: Biochemistry and Molecular Biology 146, 207–214 (2007). 10.1016/j.cbpb.2006.10.105 17150393

[b52] Zhang C., Xie L., Huang J., Chen L. & Zhang R. A novel putative tyrosinase involved in periostracum formation from the pearl oyster (*Pinctada fucata*). Biochemical and biophysical research communications 342, 632–639 (2006). 16488396 10.1016/j.bbrc.2006.01.182

[b53] Timmermans L. P. Studies on shell formation in molluscs. Netherlands Journal of Zoology 19, 413–523 (1968).

[b54] Gesualdo I., Aniello F., Branno M. & Palumbo A. Molecular cloning of a peroxidase mRNA specifically expressed in the ink gland of *Sepia officinalis*. Biochimica et Biophysica Acta (BBA)-Gene Structure and Expression 1353, 111–117 (1997). 9294004 10.1016/s0167-4781(97)00088-2

[b55] Shiro Y., Kurono M. & Morishima I. Presence of endogenous calcium ion and its functional and structural regulation in horseradish peroxidase. Journal of Biological Chemistry 261, 9382–9390 (1986). 3013887

[b56] Dosztányi Z., Csizmók V., Tompa P. & Simon I. IUPred: web server for the prediction of intrinsically unstructured regions of proteins based on estimated energy content. Bioinformatics 21, 3433–3434 (2005). 15955779 10.1093/bioinformatics/bti541

[b57] Newman A. M. & Cooper J. B. XSTREAM: A practical algorithm for identification and architecture modeling of tandem repeats in protein sequences. BMC Bioinformatics 8, 382 (2007). 17931424 10.1186/1471-2105-8-382 PMC2233649

[b58] Camacho C. . BLAST+: architecture and applications. BMC bioinformatics 10, 1 (2009). 20003500 10.1186/1471-2105-10-421 PMC2803857

[b59] Krzywinski M. . Circos: an information aesthetic for comparative genomics. Genome research 19, 1639–1645 (2009). 19541911 10.1101/gr.092759.109 PMC2752132

[b60] Letunic I., Doerks T. & Bork P. SMART: recent updates, new developments and status in 2015. Nucleic Acids Research 43, 257–260 (2015). 10.1093/nar/gku949 PMC4384020 25300481

[b61] Quevillon E. . InterProScan: protein domains identifier. Nucleic Acids Research 33, 116–120 (2005). 10.1093/nar/gki442 PMC1160203 15980438

[b62] Tamura K. . MEGA5: molecular evolutionary genetics analysis using maximum likelihood, evolutionary distance, and maximum parsimony methods. Molecular biology and evolution 28, 2731–2739 (2011). 21546353 10.1093/molbev/msr121 PMC3203626

[b63] Petersen T. N., Brunak S., von Heijne G. & Nielsen H. SignalP 4.0: discriminating signal peptides from transmembrane regions. Nature methods 8, 785–786 (2011). 21959131 10.1038/nmeth.1701

[b64] Lupas A., Van Dyke M. & Stock J. Predicting coiled coils from protein sequences. Science 252, 1162–1164 (1991). 2031185 10.1126/science.252.5009.1162

[b65] Möller S., Croning M. D. & Apweiler R. Evaluation of methods for the prediction of membrane spanning regions. Bioinformatics (Oxford, England) 17, 646–653 (2001). 10.1093/bioinformatics/17.7.646 11448883

[b66] Tellam R. L., Vuocolo T., Johnson S. E., Jarmey J. & Pearson R. D. Insect chitin synthase. European Journal of Biochemistry 267, 6025–6043 (2000). 10998064 10.1046/j.1432-1327.2000.01679.x

