# Supplementary material for: Identification of conserved proteins from diverse shell matrix proteome in Crassostrea gigas: characterization of genetic bases regulating shell formation
Source: Sci Rep. 2017 Apr 4;7:45754. doi: 10.1038/srep45754 (PMC5379566; doi:10.1038/srep45754)
Supplement: Supplementary Information [file srep45754-s1.pdf]

Identification of conserved proteins from diverse shell matrix proteome in *Crassostrea gigas*: characterization of genetic bases regulating shell formation

Dandan Feng[1], Qi Li [1,2,*], Hong Yu[1], Lingfeng Kong[1], Shaojun Du[3]

1 *Key Laboratory of Mariculture, Ministry of Education, Ocean University of China, Qingdao 266003, China；*

2 *Laboratory for Marine Fisheries Science and Food Production Processes, Qingdao National Laboratory for Marine Science and Technology*

3 *Institute of Marine and Environmental Technology, Department of Biochemistry and Molecular Biology, University of Maryland School of Medicine, Baltimore, MD, United States*

*corresponding author: qili66@ouc.edu.cn

Fig. S1 Simplified phylogeny of the ten species.

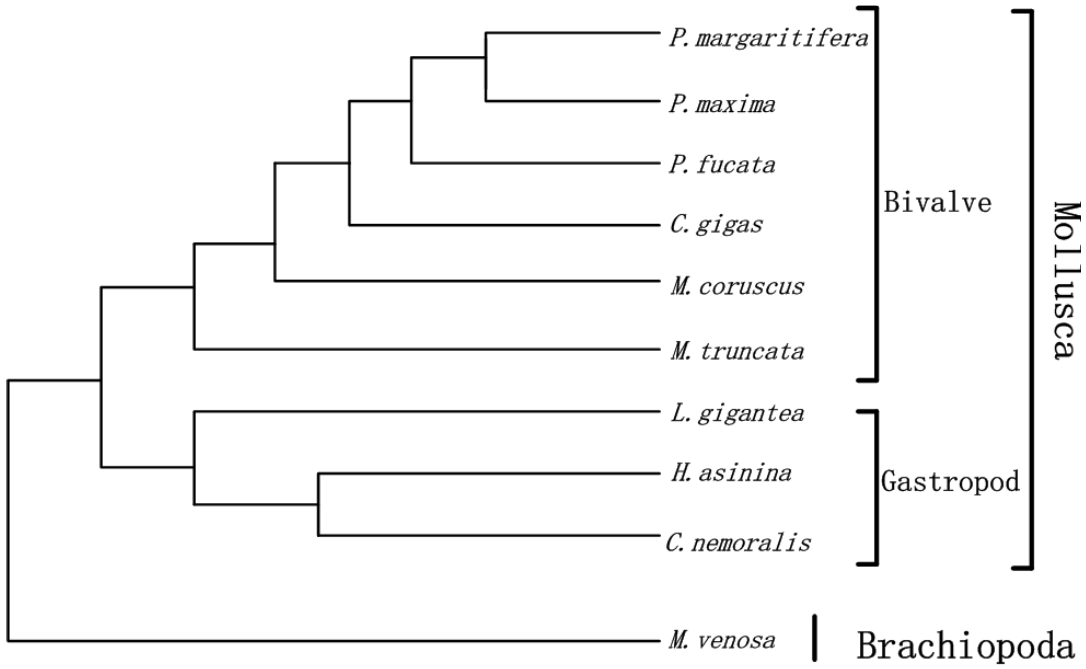

Fig. S2 Phylogenetic tree of Calmodulin related proteins in Mollusca. The tree was constructed using the NJ method, and the number at each node shows the bootstrap value.

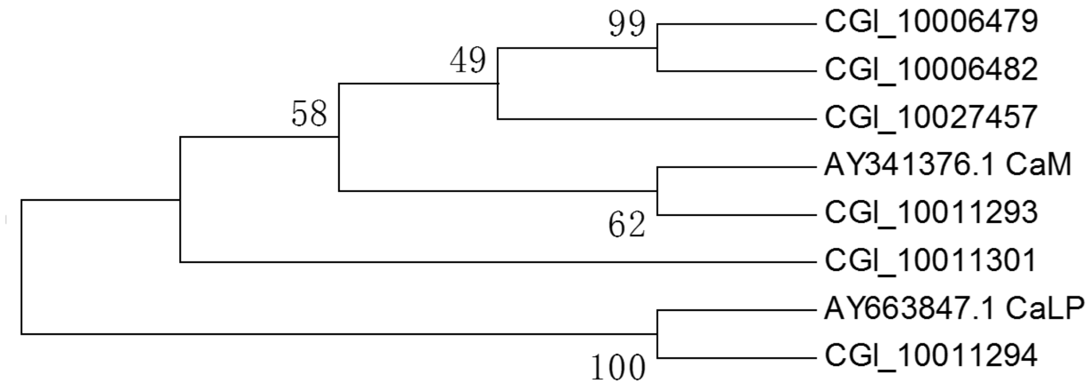

Fig. S3 Phylogenetic tree of Nacrein related proteins in Mollusca. The tree was constructed using the NJ method, and the number at each node shows the bootstrap value.

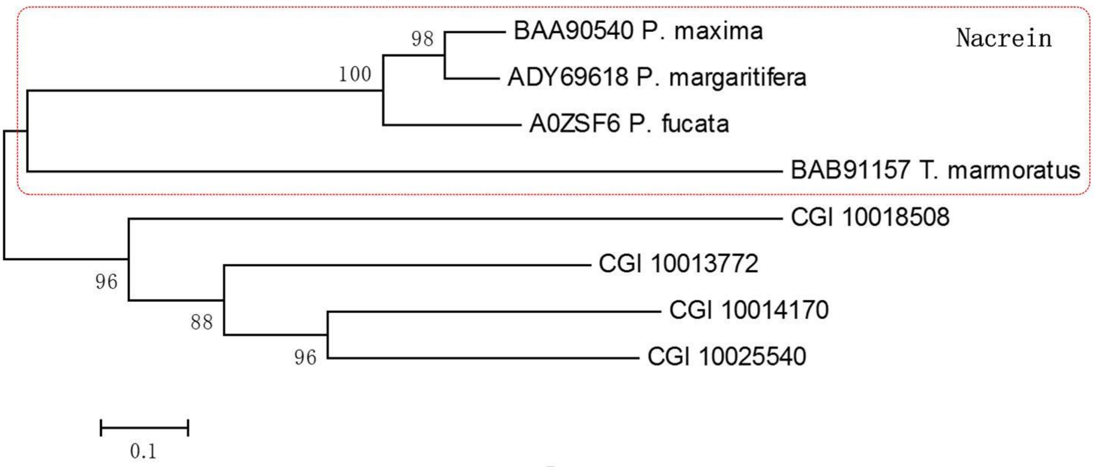

Fig. S4 Amino acid sequence alignment of EGF-ZP in Mollusca. The signal peptides were underlined. Conserved EGF-like domains are boxed, and 6 cysteine residues of each EGF-like domain that are potentially involved in disulphide bonds. Asterisks indicate the conserved cysteine residues.

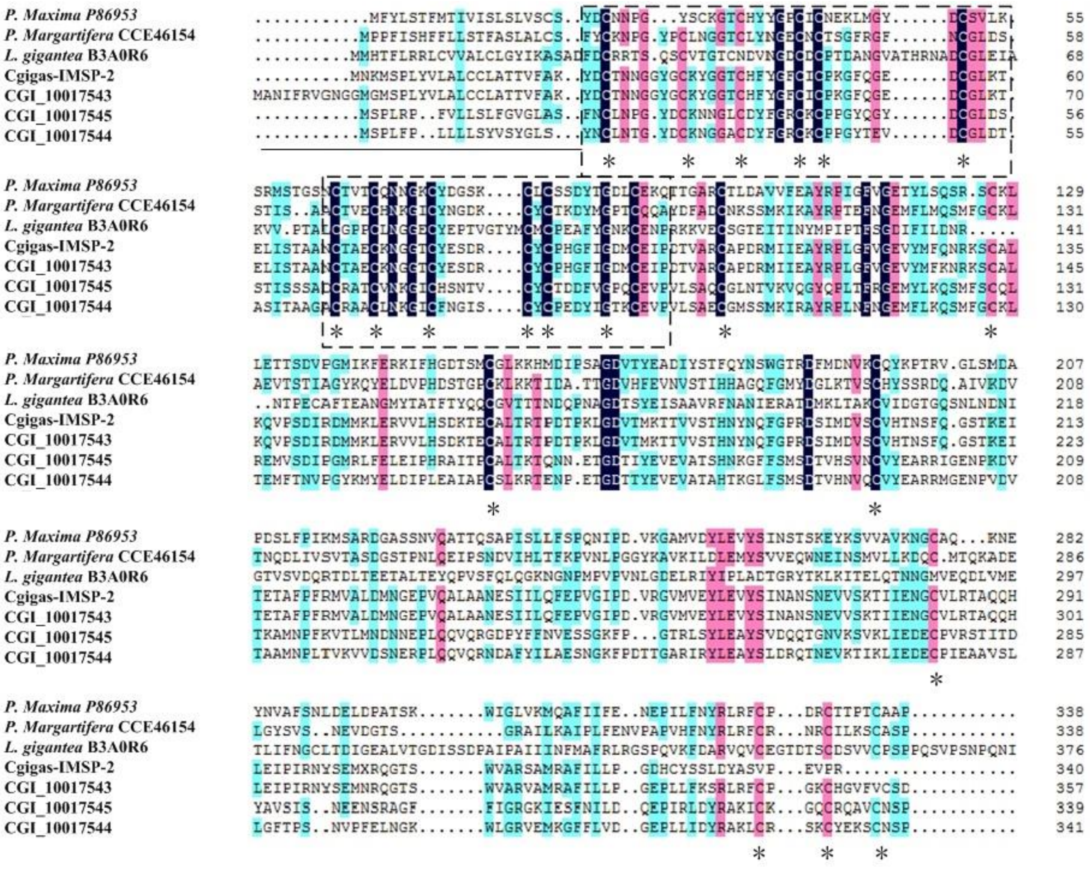

Supplementary Table S1 Disorder region and tandem repeat in the SMPs

Gene accession in 76 SMPE	Disorder region	Tandem repeat	Gene accession in 53 SMPE	Disorder region	Tandem repeat
CGI_10007002	+	-	CGI_10005425	+	−
CGI_10028037	-	-	CGI_10017544	+	−
CGI_10008376	+	+	CGI_10005749	+	−
CGI_10011913	+	-	CGI_10009194	+	−
CGI_10016430	+	-	CGI_10012743	+	−
CGI_10007556	+	+	CGI_10005424	−	−
CGI_10011015	+	-	CGI_10016964	+	+
CGI_10007857	+	+	CGI_10017545	+	−
CGI_10005424	-	-	CGI_10028014	+	−
CGI_10028414	-	-	CGI_10018175	+	+
CGI_10022840	+	-	CGI_10003000	+	−
CGI_10009634	-	+	CGI_10012348	+	+
CGI_10007530	+	-	CGI_10004227	+	−
CGI_10011916	+	-	CGI_10017543	+	−
CGI_10007753	+	-	CGI_10023765	+	−
CGI_10012348	+	+	CGI_10016965	+	+
CGI_10005425	+	-	CGI_10016397	+	+
CGI_10012353	+	-	CGI_10005096	+	+
CGI_10003000	+	-	CGI_10023928	+	−
CGI_10026599	+	-	CGI_10008375	+	+
CGI_10028286	-	-	CGI_10020612	+	+
CGI_10018174	+	+	CGI_10006505	+	+
CGI_10005220	-	-	CGI_10023851	+	−
CGI_10004228	+	-	CGI_10012352	+	+
CGI_10008434	+	+	CGI_10020756	+	−
CGI_10017544	+	-	CGI_10007857	+	+
CGI_10026605	+	+	CGI_10024229	+	+
CGI_10027091	+	+	CGI_10014170	+	−
CGI_10016016	+	+	CGI_10020608	−	−
CGI_10015567	-	-	CGI_10021716	−	−
CGI_10012352	+	+	CGI_10026603	+	+
CGI_10004227	+	-	CGI_10018174	+	+
CGI_10008711	+	-	CGI_10012353	+	−
CGI_10018173	+	+	CGI_10021733	+	−
CGI_10004704	+	+	CGI_10028414	−	−
CGI_10028014	+	-	CGI_10002445	+	−
CGI_10016999	+	+	CGI_10008434	+	+
CGI_10023446	+	+	CGI_10007021	−	−
CGI_10017398	+	-	CGI_10016430	+	−
CGI_10017543	+	-	CGI_10027091	+	+

CGI_10005096	+	+	CGI_10015700	+	−
CGI_10012419	+	-	CGI_10016015	+	−
CGI_10023928	+	-	CGI_10010359	+	+
CGI_10012743	+	-	CGI_10023446	+	+
CGI_10010359	+	+	CGI_10014161	+	−
CGI_10009988	+	-	CGI_10026600	+	−
CGI_10010526	+	-	CGI_10013462	+	−
CGI_10017085	+	-	CGI_10017398	+	−
CGI_10017087	+	-	CGI_10026390	+	+
CGI_10007514	-	-	P86784	+	+
CGI_10017545	+	-	XP.011438522	−	−
CGI_10024229	+	+	XP.011428721	+	−
CGI_10023506	+	-	XP.011453459	+	+
CGI_10005749	+	-			
CGI_10016964	+	+			
CGI_10007670	+	-			
CGI_10016965	+	+			
CGI_10004086	+	-			
CGI_10007669	+	-			
CGI_10016585	+	+			
CGI_10018176	+	+			
CGI_10018175	+	+			
CGI_10018988	+	-			
CGI_10020756	+	-			
CGI_10026432	+	-			
CGI_10006505	+	+			
CGI_10023851	+	-			
CGI_10026671	+	-			
CGI_10018952	-	-			
CGI_10002524	+	-			
CGI_10020694	+	-			
CGI_10028495	+	-			
CGI_10020232	+	-			
CGI_10005733	+	-			
CGI_10007411	-	-			
CGI_10014785	+	-			

Table S2 Top BLASTp hits returned against C. gigas queries from nine shell proteomes

<table>
<tr><th>Qurey ID</th><th>Count in Bivalve</th><th>Count all</th><th>P.fucata (e-value)</th><th>P.margaritifera (e-value)</th><th>P.maxima (e-value)</th><th>M.truncata (e-value)</th><th>M.coruscus (e-value)</th><th>L.gigantea (e-value)</th><th>H.asinina (e-value)</th><th>C.nemoralis (e-value)</th><th>M.venosa (e-value)</th><th>Description</th></tr>
<tr><td>CGI_10017544</td><td>3</td><td>4</td><td>pfu_aug1.0_853.1_22356.t1</td><td>gi|391359265|</td><td>gi|353558796|</td><td></td><td></td><td>gi|374110557|</td><td></td><td></td><td></td><td>Wnt inhibitory factor 1</td></tr>
<tr><td></td><td></td><td></td><td>2.00E-68</td><td>1.00E-83</td><td>3.00E-86</td><td></td><td></td><td>3.00E-18</td><td></td><td></td><td></td><td></td></tr>
<tr><td>CGI_10005749</td><td>0</td><td>2</td><td></td><td></td><td></td><td></td><td></td><td></td><td></td><td>contig_1188:71:538</td><td>A1256_Magellania1208_f1002380</td><td>uncharacterized protein LOC105338188 isoform X1</td></tr>
<tr><td></td><td></td><td></td><td></td><td></td><td></td><td></td><td></td><td></td><td></td><td>1.00E-41</td><td>7.00E-92</td><td></td></tr>
<tr><td>CGI_10009194</td><td>4</td><td>5</td><td>pfu_aug1.0_10759.1_31979.t1</td><td>gi|391359324|</td><td></td><td>contig02027:255:1193</td><td>gi|906541703|</td><td>gi|175684/239574|</td><td></td><td></td><td></td><td>uncharacterized protein LOC105319624 isoform X1</td></tr>
<tr><td></td><td></td><td></td><td>2.00E-117</td><td>1.00E-25</td><td></td><td>3.00E-12</td><td>3.00E-46</td><td>2.00E-14</td><td></td><td></td><td></td><td></td></tr>
<tr><td>CGI_10012743</td><td>5</td><td>6</td><td>pfu_aug1.0_16905.1_25558.t1</td><td>gi|391359338|</td><td>gi|353558891|</td><td>contig00902:31:1749</td><td>gi|906541766|</td><td></td><td></td><td></td><td>A1256_Magellania1208_r10023620</td><td>Putative tyrosinase-like protein tyr-3</td></tr>
<tr><td></td><td></td><td></td><td>3.00E-46</td><td>9.00E-46</td><td>5.00E-47</td><td>2.00E-49</td><td>4.00E-36</td><td></td><td></td><td></td><td>8.00E-17</td><td></td></tr>
<tr><td>CGI_10016964</td><td>3</td><td></td><td>pfu_aug1.0_11437.1_03076.t1</td><td>gi|391359279|</td><td></td><td></td><td>gi|824631368|</td><td></td><td></td><td></td><td></td><td>Tyrosine-protein phosphatase Lar</td></tr>
<tr><td></td><td></td><td></td><td>8.00E-94</td><td>2.00E-165</td><td></td><td></td><td>7.00E-170</td><td></td><td></td><td></td><td></td><td></td></tr>
<tr><td>CGI_10017545</td><td>3</td><td>4</td><td>pfu_aug1.0_853.1_22356.t1</td><td>gi|391359265|</td><td>gi|353558796|</td><td></td><td></td><td>gi|374110557|</td><td></td><td></td><td></td><td>Teneurin-2</td></tr>
<tr><td></td><td></td><td></td><td>4.00E-69</td><td>3.00E-76</td><td>6.00E-77</td><td></td><td></td><td>1.00E-13</td><td></td><td></td><td></td><td></td></tr>
<tr><td>CGI_10028014</td><td>4</td><td>5</td><td>pfu_aug1.0_1843.1_37145.t1</td><td>gi|391359324|</td><td></td><td>contig01866:900:1382</td><td>gi|906541739|</td><td>gi|175684/239574|</td><td></td><td></td><td></td><td>uncharacterized protein LOC105325156</td></tr>
<tr><td></td><td></td><td></td><td>2.00E-66</td><td>4.00E-45</td><td></td><td>3.00E-17</td><td>3.00E-49</td><td>2.00E-44</td><td></td><td></td><td></td><td></td></tr>
<tr><td>CGI_100</td><td>2</td><td></td><td>pfu_aug1.0_142</td><td></td><td></td><td></td><td>gi|90654</td><td></td><td></td><td></td><td></td><td>Complement C1q-like</td></tr>
</table>

03000			19.1_03462.t1				1745|					protein 2
			3.00E-10				8.00E-10					
CGI_100 12348	3	4	pfu_aug1.0_184 3.1_37145.t1			contig05 387:841: 104	gi|90654 1676|		ML5H 8			uncharacterized protein LOC105345521 isoform X1
			4.00E-09			1.00E-11	4.00E-24		3.00E-06			
CGI_100 17543	3	4	pfu_aug1.0_853 .1_22356.t1	gi|3913592 64|	gi|35355 8795|			gi|3741105 57|				Wnt inhibitory factor 1
			1.00E-48	1.00E-85	3.00E-91			3.00E-15				
CGI_100 23765	2					contig00 475:1794 :70	gi|90654 1769|					CD109 antigen-like
						0	0					
CGI_100 16965	3		pfu_aug1.0_114 37.1_03076.t1	gi|3913592 79|			gi|82463 1368|					Tyrosine-protein phosphatase Lar
			2.00E-77	2.00E-139			1.00E-146					
CGI_100 16397	5	6	pfu_aug1.0_169 05.1_25558.t1	gi|3913593 38|	gi|35355 8891|	contig00 902:31:1 749	gi|90654 1766|				A1256_Magellani a1208_r10023620	Histone-lysine N-methyltransferase MLL3
			2.00E-33	3.00E-36	4.00E-35	1.00E-47	4.00E-51				3.00E-24	
CGI_100 20612	1	1	pfu_aug1.0_750 94.1_56521.t1									hypothetical protein CGI_10020612
			3.00E-06									
CGI_100 23851	0	1						gi|3741107 50|				Peptidyl-prolyl cis-trans isomerase B
								3.00E-58				
CGI_100 12352	4	5	pfu_aug1.0_184 3.1_37145.t1	gi|3913593 24|		contig05 387:841: 104	gi|90654 1739|	gi|175684/ 239574|				Follistatin-related protein 4
			4.00E-17	1.00E-12		2.00E-18	2.00E-29	2.00E-10				

CGI_10020756	3	4		gi|391359293|	gi|353558671|		gi|906541736|		P0012N13_463			putative uncharacterized protein DDB_G0293878 isoform X1
				5.00E-15	4.00E-08		8.00E-11		1.00E-09			
CGI_10007857	2	2		gi|391359282|	gi|353558649|							Chitobiase
				7.00E-116	2.00E-110							
CGI_10014170	4	6	pfu_aug1.0_8238.1_17260.t1		gi|74826594|	contig00764:66:1418	gi|906541754|	gi|374110482|		contig_25891:609:3695		Nacrein-like protein
			7.00E-13		9.00E-18	9.00E-13	5.00E-108	1.00E-12		1.00E-10		
CGI_10026603	5	6	pfu_aug1.0_10761.1_31980.t1	gi|391738044|	gi|353558824|	contig00108:2971:632	gi|906541763|			contig_248122:627:1		Chitotriosidase-1
			7.00E-69	9.00E-76	2.00E-68	3.00E-175	1.00E-101			6.00E-20		
CGI_10012353	4	5	pfu_aug1.0_1843.1_37145.t1	gi|391359324|		contig00670:1891:32	gi|906541739|	gi|175684/239574|				hypothetical protein CGI_10012353
			6.00E-06	5.00E-10		3.00E-11	3.00E-17	4.00E-10				
CGI_10021733	1	3		gi|391359325|				gi|374110746|			A1256_Magellania1208_f20107906	Peroxidasin
				7.00E-88				7.00E-46			6.00E-93	
CGI_10028414	1					contig07764:573:280						Kielin/chordin-like protein
						8.00E-09						
CGI_10007021	1					contig07764:573:280						kielin/chordin-like protein

<table>
<tr><td></td><td></td><td></td><td></td><td></td><td></td><td>2.00E-08</td><td></td><td></td><td></td><td></td><td></td><td></td></tr>
<tr><td>CGI_10016430</td><td>3</td><td>3</td><td>pfu_aug1.0_75094.1_56521.t1</td><td>gi|391359262|</td><td>gi|353558894|</td><td></td><td></td><td></td><td></td><td></td><td></td><td>hypothetical protein CGI_10016430</td></tr>
<tr><td></td><td></td><td></td><td>9.00E-29</td><td>1.00E-23</td><td>1.00E-23</td><td></td><td></td><td></td><td></td><td></td><td></td><td></td></tr>
<tr><td>CGI_10010359</td><td>4</td><td>5</td><td>pfu_aug1.0_1843.1_37145.t1</td><td>gi|391359317|</td><td></td><td>contig05387:841:104</td><td>gi|906541739|</td><td>gi|374253727|</td><td></td><td></td><td></td><td>hypothetical protein CGI_10010359</td></tr>
<tr><td></td><td></td><td></td><td>7.00E-20</td><td>1.00E-32</td><td></td><td>2.00E-22</td><td>1.00E-30</td><td>2.00E-12</td><td></td><td></td><td></td><td></td></tr>
<tr><td>CGI_10014161</td><td>0</td><td>1</td><td></td><td></td><td></td><td></td><td></td><td></td><td></td><td></td><td>A1256_Magellania1208_r30126366</td><td>Chymotrypsin-like serine proteinase</td></tr>
<tr><td></td><td></td><td></td><td></td><td></td><td></td><td></td><td></td><td></td><td></td><td></td><td>3.00E-25</td><td></td></tr>
<tr><td>CGI_10026600</td><td>5</td><td>5</td><td>pfu_aug1.0_10761.1_31980.t1</td><td>gi|391738044|</td><td>gi|353558824|</td><td>contig00108:2971:632</td><td>gi|906541763|</td><td></td><td></td><td></td><td></td><td>Chitotriosidase-1</td></tr>
<tr><td></td><td></td><td></td><td>2.00E-142</td><td>9.00E-176</td><td>4.00E-148</td><td>9.00E-64</td><td>1.00E-42</td><td></td><td></td><td></td><td></td><td></td></tr>
<tr><td>CGI_10013462</td><td>0</td><td>1</td><td></td><td></td><td></td><td></td><td></td><td>gi|174428/239129|</td><td></td><td></td><td></td><td>hypothetical protein CGI_10013462</td></tr>
<tr><td></td><td></td><td></td><td></td><td></td><td></td><td></td><td></td><td>3.00E-42</td><td></td><td></td><td></td><td></td></tr>
</table>

Table S3 List of shell matrix proteins used for BLAST

Gene name	Species	BLASTp				BLASTn			
		Access No.	Best Subject ID	Score	E-value	Access No.	Best Subject ID	Score	E-value
Cgtyr1	*C. gigas*	AGZ15753.1	CGI_10007793	1352	0.0E+00	KC878467	CGI_10007793	2418	0
CgTyr2	*C. gigas*	KF695384	CGI_10011913	1415	0.0E+00	KF695384.1	CGI_10011913	2702	0
Cgigas-IMSP-2	*C. gigas*					AM868448; AM864770	CGI_10017543	663	0.0E+00
SPARC	*P.fucata*	BAK22656	CGI_10005088	245	2.0E-65	AB600273.1	No hit		
PFMG2	*P.fucata*	AAZ76256	CGI_10006016	204	3.0E-53	DQ104256.1	CGI_10006016	64	2.00E-08
CaLP	*P.fucata*	AAV73912	CGI_10006479	204	2.0E-53	AY663847	CGI_10006482	218	1.0E-57
CaM	*P.fucata*	AAQ20043.1	CGI_10006479	290	3.0E-79	AY341376.1	CGI_10011293	176	9.0E-43
Chitin synthase	*P.fucata*	BAF73720	CGI_10009438	3238	0.0E+00	AB290881.1	CGI_10009438	250	3.0E-85
Nacrein	*P.fucata*	BAA11940	CGI_10014170	93	4.0E-19	D83523.1	No hit		
Pif	*P.fucata*	BAH97338	CGI_10014497	190	4.0E-48	AB236929.1	No hit		
ACCBP	*P.fucata*	ABF13208	CGI_10025575	115	1.0E-25	DQ473430.1	No hit		
PFMG9	*P.fucata*	AAZ22318	CGI_10010153	146	1.0E-35	DQ116436.1	No hit		
Pfn44	*P.fucata*	AGG35567	CGI_10014775	130	1.0E-30				
BMSP	*M. galloprovincialis*	BAK86420	CGI_10009194	1415	0.0E+00				
Chitobiase	*P. margaritifera*	CCE46157	CGI_10007857	368	1.0E-101	HE610383.1			
Fibronectin 1	*P. margaritifera*	CCE46158	CGI_10016964	399	1.0E-111	HE610379.1			
Fibronectin 3	*P. margaritifera*	CCE46159	CGI_10016964	493	1.0E-139	HE610385.1			
EFG-like1	*P. margaritifera*	CCE46153	CGI_10017543	263	9.0E-71	HE610379.1			
EFG-like2	*P. margaritifera*	CCE46154	CGI_10017544	254	4.0E-68	HE610380.1			
Peroxidase	*P. margaritifera*	CCE46168	CGI_10023200	424	1.0E-118	HE610394.1			
Clp3	*P. margaritifera*	CCE46156	CGI_10026599	541	1.0E-154	HE610382.1			
CopAmOx	*P. margaritifera*	CCE46164	CGI_10028897	718	0.0E+00	HE610390.1			
Ependymin-like	*P.fucata*	pfu_aug1.0_725_00459	No hit			pfu_aug1.0_725.1_00459.t1	No hit		
N16	*P.fucata*	BAA83732	No hit			AB023067.1	No hit		
N19	*P.fucata*	BAK57305	No hit			AB649990.1	No hit		
Pfu000096	*P.fucata*	BAK40911	No hit			AB635374.1	No hit		
KRMP-1	*P.fucata*	BAH78717	No hit			AB507411.1	No hit		

KRMP-3	*P.fucata*	BAH78729	No hit			AB507423.1	No hit		
KRMP-4	*P.fucata*	BAH78734	No hit			AB507428.1	No hit		
MSI60	*P.fucata*	BAA20466	No hit			D86074.1	No hit		
MSI60-related	*P.fucata*	BAL42250	No hit			AB689024.1	No hit		
Prisilkin-39	*P.fucata*	ACJ06766	No hit			EU921665.1	No hit		
Prismalin-14	*P.fucata*	BAD27406	No hit			AB159512.1	No hit		
Prismin	*P.fucata*	BAG28185	No hit			AB433980.2	No hit		
SGMP1	*P.fucata*	BAL42249	No hit			AB689023.1	No hit		
shematrin-1	*P.fucata*	BAE93433	No hit			AB244419.1	No hit		
shematrin-2	*P.fucata*	BAE93434	No hit			AB244420.1	No hit		
shematrin-3	*P.fucata*	BAE93435	No hit			AB244421.1	No hit		
shematrin-4	*P.fucata*	BAE93436	No hit			AB244422.1	No hit		
shematrin-5	*P.fucata*	BAE93437	No hit			AB244423.1	No hit		
shematrin-6	*P.fucata*	BAE93438	No hit			AB244424.1	No hit		
shematrin-7	*P.fucata*	BAE93439	No hit			AB244425.1	No hit		
PFMG1	*P.fucata*	AAZ76260	No hit			DQ104260.1	No hit		
PFMG3	*P.fucata*	AAZ76257	No hit			DQ104257.1	No hit		
PFMG4	*P.fucata*	AAZ76258	No hit			DQ104258.1	No hit		
PFMG5	*P.fucata*	AAZ76259	No hit			DQ104259.1	No hit		
PFMG8	*P.fucata*	AAZ76262	No hit			DQ104262.1	No hit		
PFMG10	*P.fucata*	AAZ22319	No hit			DQ116437.1	No hit		
PFMG11	*P.fucata*	AAZ22320	No hit			DQ116438.1	No hit		
PFMG12	*P.fucata*	AAZ22321	No hit			DQ116439.1	No hit		
Aspein	*P.fucata*	BAD00044	No hit			pfu_aug1.0_465.1_00315.t1	No hit		
PfN23	*P.fucata*	AFJ19278	No hit						
Mp10	*P. margaritifera*	CCE46148	No hit						
Alveoline-like	*P. margaritifera*	CCE46147	No hit						
PTIMP1	*P. margaritifera*	CCE46165	No hit						
PTIMP2	*P. margaritifera*	CCE46173	No hit						
PTIMP3	*P. margaritifera*	CCE46174	No hit						

Table S4 Details of the global BLASTp results

Query_id	Query_length	Query_start	Query_end	Subject_id	Subject _length	Subject _start	Subject_end	Match_length	Subject_coverage	Identity	Eval ue	Bit-score
CGI_10017544	362	1	362	gi|353558796|sp|P86954.1|ELDP2_PINMA RecName: Full=EGF-like domain-containing protein 2; Flags: Precursor [Pmaxima15]	357	5	357	365	98.88	41.92	3.00 E-86	263
CGI_10017544	362	7	362	gi|391359265|sp|H2A0L3.1|ELDP2_PINMG RecName: Full=EGF-like domain containing protein 2; Flags: Precursor [Pinctada margaritifera9]	359	9	359	359	97.77	40.67	1.00 E-83	256
CGI_10017544	362	1	353	pfu_aug1.0_853.1_22356.t1[P fucata56]	363	1	354	361	97.52	36.84	2.00 E-68	217
CGI_10017544	362	5	350	gi|391359264|sp|H2A0L2.1|ELDP1_PINMG RecName: Full=EGF-like domain containing protein 1; Flags: Precursor [Pinctada margaritifera8]	348	7	347	354	97.99	30.23	1.00 E-56	186
CGI_10017544	362	5	341	gi|353558795|sp|P86953.1|ELDP1_PINMA RecName: Full=EGF-like domain-containing protein 1; Flags: Precursor[Pmaxima14]	348	7	338	338	95.4	30.18	2.00 E-54	180
CGI_10017544	362	8	198	gi|374110557|sp|B3A0R6.1|ELDP1_LOTGI RecName: Full=EGF-like domain-containing protein 1; AltName: Full=Uncharacterized shell protein 17; Short=LUSP-17; Flags: Precursor[Lgigantea14]	513	14	208	208	38.01	27.88	3.00 E-18	80.1
CGI_10017544	362	7	198	gi|374110558|sp|B3A0S3.1|ELDP2_LOTGI RecName: Full=EGF-like domain-containing protein 2; AltName: Full=Uncharacterized shell protein 24; Short=LUSP-24; Flags: Precursor[Lgigantea15]	489	13	209	209	40.29	26.79	7.00 E-17	75.9
CGI_10005749	639	27	533	A1256_Magellania1208_f1002380[Mvenosa1] 303	1018	155	661	518	49.8	36.68	7.00 E-92	
CGI_10005749	639	18	544	A1256_Magellania1208_r20087389[Mvenosa47] 287	963	81	627	568	56.8	33.98	3.00 E-86	
CGI_10005749	639	150	306	lcl|ORF1_contig_1188:71:538[Cnemoralis14] 144	156	1	156	159	100	46.54	1.00 E-41	

CGI_10009194	2516	1116	1697	pfu_aug1.0_10759.1_31979.t1[P fucata37]	901	110	677	623	63.04	37.88	2.00 E-117	393
CGI_10009194	2516	2173	2481	pfu_aug1.0_10759.1_31979.t1[P fucata37]	901	527	879	355	39.18	33.24	9.00 E-40	155
CGI_10009194	2516	1575	1932	pfu_aug1.0_10759.1_31979.t1[P fucata37]	901	294	631	366	37.51	29.23	1.00 E-39	155
CGI_10009194	2516	1694	2071	pfu_aug1.0_10759.1_31979.t1[P fucata37]	901	285	633	393	38.73	25.95	8.00 E-33	133
CGI_10009194	2516	866	917	pfu_aug1.0_10759.1_31979.t1[P fucata37]	901	56	107	52	5.77	57.69	5.00 E-17	81.6
CGI_10009194	2516	1837	2119	pfu_aug1.0_10759.1_31979.t1[P fucata37]	901	294	567	292	30.41	24.66	2.00 E-13	69.7
CGI_10009194	2516	25	178	pfu_aug1.0_287428.1_50307.t1[P fucata41]	155	1	155	155	100	76.77	1.00 E-75	246
CGI_10009194	2516	236	389	pfu_aug1.0_287428.1_50307.t1[P fucata41]	155	1	155	155	100	50.97	3.00 E-47	165
CGI_10009194	2516	448	600	pfu_aug1.0_287428.1_50307.t1[P fucata41]	155	2	155	154	99.35	52.6	2.00 E-46	162
CGI_10009194	2516	680	831	pfu_aug1.0_287428.1_50307.t1[P fucata41]	155	1	151	152	97.42	35.53	3.00 E-25	101
CGI_10009194	2516	967	1114	pfu_aug1.0_21286.1_69411.t1[P fucata64]	152	1	151	151	99.34	56.29	2.00 E-54	185
CGI_10009194	2516	230	364	gi|906541703|gb|AKS48145.1| collagen-like protein-1, partial [Mytilus coruscus23]	158	22	156	135	85.44	60	3.00 E-46	162
CGI_10009194	2516	441	575	gi|906541703|gb|AKS48145.1| collagen-like protein-1, partial [Mytilus coruscus23]	158	22	156	135	85.44	51.11	8.00 E-37	135
CGI_10009194	2516	20	153	gi|906541703|gb|AKS48145.1| collagen-like protein-1, partial [Mytilus coruscus23]	158	23	156	134	84.81	47.01	2.00 E-31	119
CGI_10009194	2516	674	809	gi|906541703|gb|AKS48145.1| collagen-like protein-1, partial [Mytilus coruscus23]	158	22	156	136	85.44	32.35	4.00 E-20	86.3
CGI_10009194	2516	857	1104	gi|906541739|gb|AKS48157.1| sushi-like protein [Mytilus coruscus35]	844	473	719	257	29.27	29.57	5.00 E-30	124

CGI_10009194	2516	445	618	gi|906541739|gb|AKS48157.1| sushi-like protein [Mytilus coruscus35]	844	32	206	175	20.73	39.43	1.00 E-28	119
CGI_10009194	2516	233	407	gi|906541739|gb|AKS48157.1| sushi-like protein [Mytilus coruscus35]	844	31	206	176	20.85	34.09	6.00 E-28	117
CGI_10009194	2516	23	204	gi|906541739|gb|AKS48157.1| sushi-like protein [Mytilus coruscus35]	844	32	216	185	21.92	33.51	6.00 E-24	104
CGI_10009194	2516	678	852	gi|906541739|gb|AKS48157.1| sushi-like protein [Mytilus coruscus35]	844	32	206	176	20.73	24.43	1.00 E-13	70.5
CGI_10009194	2516	2391	2508	gi|906541739|gb|AKS48157.1| sushi-like protein [Mytilus coruscus35]	844	715	833	123	14.1	29.27	7.00 E-09	55.1
CGI_10009194	2516	788	1107	gi|906541679|gb|AKS48137.1| matrix protein-1 [Mytilus coruscus15]	635	188	515	339	51.65	30.68	2.00 E-27	114
CGI_10009194	2516	854	1103	pfu_aug1.0_1843.1_37145.t1[P fucata52]	1923	1553	1802	254	13	31.89	7.00 E-27	114
CGI_10009194	2516	857	1104	gi|391359324|sp|H2A0N4.1|PIF_PINMG RecName: Full=Protein PIF; Contains: RecName: Full=Protein Pif97; Contains: RecName: Full=Protein Pif80; AltName: Full=Aragonite-binding protein;Flags: Precursor [Pinctada margaritifera20]	1014	259	538	284	27.61	27.11	1.00 E-25	110
CGI_10009194	2516	444	621	gi|391359324|sp|H2A0N4.1|PIF_PINMG RecName: Full=Protein PIF; Contains: RecName: Full=Protein Pif97; Contains: RecName: Full=Protein Pif80; AltName: Full=Aragonite-binding protein;Flags: Precursor [Pinctada margaritifera20]	1014	28	204	179	17.46	31.84	3.00 E-19	89
CGI_10009194	2516	22	203	gi|391359324|sp|H2A0N4.1|PIF_PINMG RecName: Full=Protein PIF; Contains: RecName: Full=Protein Pif97; Contains: RecName: Full=Protein Pif80; AltName: Full=Aragonite-binding protein;Flags: Precursor [Pinctada margaritifera20]	1014	28	208	183	17.85	30.6	6.00 E-19	88.2
CGI_10009194	2516	233	414	gi|391359324|sp|H2A0N4.1|PIF_PINMG RecName: Full=Protein PIF; Contains: RecName: Full=Protein Pif97; Contains: RecName: Full=Protein Pif80;	1014	28	208	184	17.85	30.98	9.00 E-17	80.9

				AltName: Full=Aragonite-binding protein;Flags: Precursor [Pinctada margaritifera20]								
CGI_10009194	2516	677	854	gi|391359324|sp|H2A0N4.1|PIF_PINMG RecName: Full=Protein PIF; Contains: RecName: Full=Protein Pif97; Contains: RecName: Full=Protein Pif80; AltName: Full=Aragonite-binding protein;Flags: Precursor [Pinctada margaritifera20]	1014	28	203	179	17.36	25.14	2.00 E-11	63.5
CGI_10009194	2516	18	411	gi|906541694|gb|AKS48142.1| collagen-like protein-2 [Mytilus coruscus20]	453	32	442	424	90.73	27.36	1.00 E-24	103
CGI_10009194	2516	229	644	gi|906541694|gb|AKS48142.1| collagen-like protein-2 [Mytilus coruscus20]	453	32	453	444	93.16	28.83	1.00 E-23	100
CGI_10009194	2516	440	854	gi|906541694|gb|AKS48142.1| collagen-like protein-2 [Mytilus coruscus20]	453	32	440	438	90.29	23.06	1.00 E-09	57
CGI_10009194	2516	22	199	pfu_aug1.0_220503.1_50183.t1[P fucata71]	204	3	179	179	86.76	29.05	2.00 E-18	82.4
CGI_10009194	2516	444	621	pfu_aug1.0_220503.1_50183.t1[P fucata71]	204	3	179	179	86.76	27.93	1.00 E-17	80.1
CGI_10009194	2516	233	410	pfu_aug1.0_220503.1_50183.t1[P fucata71]	204	3	179	180	86.76	28.33	1.00 E-16	76.6
CGI_10009194	2516	677	827	pfu_aug1.0_220503.1_50183.t1[P fucata71]	204	3	151	152	73.04	28.29	2.00 E-11	60.1
CGI_10009194	2516	782	855	pfu_aug1.0_17316.1_18451.t1[P fucata40]	82	9	82	74	90.24	47.3	6.00 E-18	77.8
CGI_10009194	2516	126	198	pfu_aug1.0_17316.1_18451.t1[P fucata40]	82	9	81	73	89.02	35.62	2.00 E-11	58.9
CGI_10009194	2516	548	620	pfu_aug1.0_17316.1_18451.t1[P fucata40]	82	9	81	73	89.02	38.36	2.00 E-11	58.9
CGI_10009194	2516	337	409	pfu_aug1.0_17316.1_18451.t1[P fucata40]	82	9	81	73	89.02	36.99	1.00 E-10	56.2
CGI_10009194	2516	853	1020	gi|175684/239574|PA_LOTGI RecName:3 peritrophin-A/CBM_14 AltName: Full=Uncharacterized shell protein 20; Short=LUSP-20[Lgigantea5]	716	381	556	178	24.58	27.53	2.00 E-14	73.2

CGI_10009194	2516	2392	2507	gi|374253727|sp|B3A0P4.1|USP26_LOTGI RecName: Full=Uncharacterized shell protein 26; AltName: Full=BMSP-like protein [Lgigantea36]	173	50	161	119	64.74	31.09	2.00 E-14	69.7
CGI_10009194	2516	311	409	lcl|ORF3_Mya_contig02027:255:1193 [Mya truncata2]	312	11	113	103	33.01	36.89	3.00 E-12	64.7
CGI_10009194	2516	522	616	lcl|ORF3_Mya_contig02027:255:1193 [Mya truncata2]	312	11	111	101	32.37	34.65	6.00 E-11	60.5
CGI_10009194	2516	100	242	lcl|ORF3_Mya_contig02027:255:1193 [Mya truncata2]	312	11	144	147	42.95	29.25	1.00 E-09	56.6
CGI_10009194	2516	18	123	pfu_aug1.0_170.1_36333.t1[P fucata32]	178	37	146	112	61.8	33.93	8.00 E-12	61.2
CGI_10009194	2516	440	533	pfu_aug1.0_170.1_36333.t1[P fucata32]	178	37	128	94	51.69	36.17	8.00 E-10	55.1
CGI_10009194	2516	229	323	pfu_aug1.0_170.1_36333.t1[P fucata32]	178	37	129	97	52.25	32.99	7.00 E-07	46.2
CGI_10009194	2516	331	416	pfu_aug1.0_12760.1_53855.t1[P fucata48]	400	28	113	86	21.5	31.4	5.00 E-10	57.8
CGI_10009194	2516	122	219	pfu_aug1.0_12760.1_53855.t1[P fucata48]	400	30	115	98	21.5	33.67	2.00 E-08	52.8
CGI_10009194	2516	543	634	pfu_aug1.0_12760.1_53855.t1[P fucata48]	400	29	118	92	22.5	30.43	6.00 E-07	48.1
CGI_10009194	2516	875	977	lcl|ORF1_Mya_contig00894:20:1558 [Mya truncata8]	512	9	117	109	21.29	27.52	6.00 E-10	58.2
CGI_10009194	2516	875	977	lcl|ORF12_Mya_contig00629:1875:85 [Mya truncata12]	596	66	181	117	19.46	32.48	1.00 E-08	54.3
CGI_10009194	2516	930	989	lcl|ORF1_Mya_contig01082:16:1497 [Mya truncata6]	493	106	161	62	11.36	40.32	8.00 E-08	51.2
CGI_10009194	2516	875	1049	lcl|ORF1_Mya_contig01082:16:1497 [Mya truncata6]	493	113	282	183	34.48	23.5	7.00 E-06	45.1
CGI_10009194	2516	838	1088	lcl|ORF9_Mya_contig00670:1891:32 [Mya truncata11]	619	179	441	289	42.49	23.53	5.00 E-07	48.9
CGI_10009194	2516	858	977	lcl|ORF9_Mya_contig00670:1891:32 [Mya truncata11]	619	71	198	133	20.68	28.57	1.00 E-06	47.8

CGI_10009194	2516	1007	1103	lcl|ORF2_Mya_contig05387:841:104 [Mya truncata50]	245	12	104	104	37.96	27.88	1.00 E-06	46.2
CGI_10009194	2516	2405	2507	pfu_aug1.0_954.1_58317.t1[P fucata70]	160	60	159	108	62.5	31.48	2.00 E-06	44.3
CGI_10009194	2516	886	977	lcl|ORF8_Mya_contig01412:1442:3 [Mya truncata5]	480	7	102	97	20	29.9	8.00 E-06	44.7
CGI_10012743	1045	135	430	lcl|ORF1_Mya_contig00902:31:1749 [Mya truncata7]	573	69	371	311	52.88	37.3	2.00 E-49	180
CGI_10012743	1045	826	973	lcl|ORF1_Mya_contig00902:31:1749 [Mya truncata7]	573	430	573	154	25.13	25.97	3.00 E-09	54.7
CGI_10012743	1045	119	431	gi|353558891|sp|P86952.1|TYRO_PINMA RecName: Full=Tyrosinase-like protein; AltName: Full=Tyrosinase-2; Flags: Precursor[Pmaxima17]	456	77	402	333	71.49	33.63	5.00 E-47	171
CGI_10012743	1045	114	431	pfu_aug1.0_16905.1_25558.t1[P fucata18]	442	50	381	348	75.11	32.76	3.00 E-46	168
CGI_10012743	1045	119	431	gi|391359338|sp|H2A0L1.1|TYRO2_PINMG RecName: Full=Tyrosinase-like protein 2; AltName: Full=Tyrosinase 2; Flags: Precursor[Pinctada margaritifera27]	456	77	402	335	71.49	33.73	9.00 E-46	167
CGI_10012743	1045	180	431	pfu_aug1.0_12145.1_17832.t1[P fucata16]	315	1	268	273	85.08	33.7	1.00 E-36	137
CGI_10012743	1045	43	448	gi|906541766|gb|AKS48166.1| tyrosinase-like protein-1, partial [Mytilus coruscus44]	578	1	394	420	68.17	27.62	4.00 E-36	139
CGI_10012743	1045	116	432	gi|391359337|sp|H2A0L0.1|TYRO1_PINMG RecName: Full=Tyrosinase-like protein 1; AltName: Full=Tyrosinase 1; Flags: Precursor[Pinctada margaritifera26]	492	76	392	330	64.43	28.79	7.00 E-31	122
CGI_10012743	1045	182	430	pfu_aug1.0_3212.1_37533.t1[P fucata15]	430	36	295	268	60.47	31.34	2.00 E-26	108
CGI_10012743	1045	188	430	pfu_aug1.0_10251.1_39018.t1[P fucata8]	390	1	254	262	65.13	31.3	4.00 E-24	100
CGI_10012743	1045	113	433	pfu_aug1.0_21093.1_62062.t1[P fucata17]	477	86	381	343	62.05	25.66	2.00 E-18	83.6

CGI_10012743	1045	239	430	A1256_Magellania1208_r10023620[Mvenosa34] 77.8	376	1	207	214	55.05	28.5	8.00 E-17	
CGI_10016964	813	7	581	gi|824631368|gb|AKI87977.1| shell protein-6 [Mytilus coruscus6]	606	2	579	580	95.38	49.31	7.00 E-170	503
CGI_10016964	813	19	585	gi|391359279|sp|H2A0L8.1|FND2_PINMG RecName: Full=Fibronectin type III domain-containing protein 2; Flags: Precursor[Pinctada margaritifera11]	624	33	614	588	93.27	47.11	2.00 E-165	493
CGI_10016964	813	22	601	gi|391359278|sp|H2A0L7.1|FND1_PINMG RecName: Full=Fibronectin type III domain-containing protein 1; Flags: Precursor[Pinctada margaritifera10]	754	160	748	594	78.12	40.57	1.00 E-134	417
CGI_10016964	813	134	498	pfu_aug1.0_11437.1_03076.t1[P fucata47]	392	8	390	386	97.7	41.71	8.00 E-94	297
CGI_10016964	813	105	284	pfu_aug1.0_11437.1_03076.t1[P fucata47]	392	83	275	203	49.23	26.11	5.00 E-08	50.1
CGI_10016964	813	116	298	pfu_aug1.0_11437.1_03076.t1[P fucata47]	392	205	387	188	46.68	25	2.00 E-06	44.7
CGI_10016964	813	21	299	pfu_aug1.0_13143.1_39453.t1[P fucata49]	306	13	303	293	95.1	43	9.00 E-56	190
CGI_10016964	813	307	448	pfu_aug1.0_155246.1_57288.t1[P fucata50]	144	2	144	143	99.31	50.35	8.00 E-43	148
CGI_10016964	813	120	245	pfu_aug1.0_155246.1_57288.t1[P fucata50]	144	11	138	131	88.89	29.01	5.00 E-06	41.2
CGI_10016964	813	170	303	pfu_aug1.0_9430.1_38910.t1[P fucata59]	165	30	162	134	80.61	43.28	1.00 E-31	117
CGI_10016964	813	514	586	pfu_aug1.0_154829.1_06667.t1[P fucata60]	127	2	74	73	57.48	64.38	6.00 E-28	105
CGI_10016964	813	196	487	gi|906541688|gb|AKS48140.1| twitchin-like protein-1 [Mytilus coruscus18]	3229	754	1046	299	9.07	22.74	6.00 E-09	53.9
CGI_10016964	813	261	535	gi|906541688|gb|AKS48140.1| twitchin-like protein-1 [Mytilus coruscus18]	3229	1194	1495	314	9.35	25.48	3.00 E-07	48.1
CGI_10016964	813	199	327	gi|906541688|gb|AKS48140.1| twitchin-like protein-1 [Mytilus coruscus18]	3229	1349	1482	138	4.15	29.71	8.00 E-07	46.6

CGI_10016964	813	162	327	gi|906541688|gb|AKS48140.1| twitchin-like protein-1 [Mytilus coruscus18]	3229	224	393	177	5.26	27.12	1.00 E-06	46.2
CGI_10016964	813	209	488	gi|906541688|gb|AKS48140.1| twitchin-like protein-1 [Mytilus coruscus18]	3229	1258	1545	305	8.92	25.9	2.00 E-06	45.1
CGI_10016964	813	101	341	gi|906541688|gb|AKS48140.1| twitchin-like protein-1 [Mytilus coruscus18]	3229	565	803	261	7.4	24.9	6.00 E-06	43.9
CGI_10017545	360	7	360	gi|353558796|sp|P86954.1|ELDP2_PINMA RecName: Full=EGF-like domain-containing protein 2; Flags: Precursor [Pmaxima15]	357	9	357	359	97.76	37.6	6.00 E-77	239
CGI_10017545	360	7	360	gi|391359265|sp|H2A0L3.1|ELDP2_PINMG RecName: Full=EGF-like domain containing protein 2; Flags: Precursor [Pinctada margaritifera9]	359	8	359	360	98.05	37.5	3.00 E-76	237
CGI_10017545	360	1	352	pfu_aug1.0_853.1_22356.t1[P fucata56]	363	1	355	360	97.8	35.83	4.00 E-69	219
CGI_10017545	360	19	339	gi|353558795|sp|P86953.1|ELDP1_PINMA RecName: Full=EGF-like domain-containing protein 1; Flags: Precursor[Pmaxima14]	348	20	338	326	91.67	32.52	2.00 E-56	185
CGI_10017545	360	8	345	gi|391359264|sp|H2A0L2.1|ELDP1_PINMG RecName: Full=EGF-like domain containing protein 1; Flags: Precursor [Pinctada margaritifera8]	348	10	344	343	96.26	31.2	2.00 E-55	183
CGI_10017545	360	18	345	gi|374110557|sp|B3A0R6.1|ELDP1_LOTGI RecName: Full=EGF-like domain-containing protein 1; AltName: Full=Uncharacterized shell protein 17; Short=LUSP-17; Flags: Precursor[Lgigantea14]	513	23	370	365	67.84	21.64	1.00 E-13	65.9
CGI_10017545	360	4	352	gi|374110558|sp|B3A0S3.1|ELDP2_LOTGI RecName: Full=EGF-like domain-containing protein 2; AltName: Full=Uncharacterized shell protein 24; Short=LUSP-24; Flags: Precursor[Lgigantea15]	489	6	379	389	76.48	20.05	2.00 E-11	59.3
CGI_10028014	1158	771	1151	pfu_aug1.0_1843.1_37145.t1[P fucata52]	1923	1557	1920	383	18.93	35.77	2.00 E-66	240
CGI_10028014	1158	747	1151	gi|906541739|gb|AKS48157.1| sushi-like protein [Mytilus coruscus35]	844	450	840	415	46.33	28.92	3.00 E-49	183

CGI_10028014	1158	589	723	gi|906541739|gb|AKS48157.1| sushi-like protein [Mytilus coruscus35]	844	32	212	181	21.45	22.1	2.00 E-09	55.8
CGI_10028014	1158	589	1033	gi|391359324|sp|H2A0N4.1|PIF_PINMG RecName: Full=Protein PIF; Contains: RecName: Full=Protein Pif97; Contains: RecName: Full=Protein Pif80; AltName: Full=Aragonite-binding protein;Flags: Precursor [Pinctada margaritifera20]	1014	29	539	520	50.39	25.77	4.00 E-45	171
CGI_10028014	1158	771	1150	gi|175684/239574|PA_LOTGI RecName:3 peritrophin-A/CBM_14 AltName: Full=Uncharacterized shell protein 20; Short=LUSP-20[Lgigantea5]	716	386	712	386	45.67	26.68	2.00 E-44	166
CGI_10028014	1158	739	1152	gi|906541679|gb|AKS48137.1| matrix protein-1 [Mytilus coruscus15]	635	231	633	418	63.46	27.75	2.00 E-39	150
CGI_10028014	1158	897	1151	gi|391359317|sp|H2A0M0.1|NRP_PINMG RecName: Full=Asparagine-rich protein; AltName: Full=Prism uncharacterized shell protein 1; Short=PUSP1; Flags: Precursor[Pinctada margaritifera19]	686	427	679	268	36.88	26.87	7.00 E-22	95.5
CGI_10028014	1158	997	1149	lcl|ORF6_Mya_contig01866:900:1382 [Mya truncata3]	161	1	161	162	100	30.86	3.00 E-17	76.6
CGI_10028014	1158	934	1033	pfu_aug1.0_13237.1_61173.t1[P fucata29]	177	66	167	102	57.63	39.22	8.00 E-16	72.4
CGI_10028014	1158	779	869	pfu_aug1.0_10759.1_31979.t1[P fucata37]	901	56	140	91	9.43	28.57	3.00 E-11	61.6
CGI_10028014	1158	766	893	lcl|ORF9_Mya_contig00670:1891:32 [Mya truncata11]	619	66	201	138	21.97	31.16	7.00 E-11	60.1
CGI_10028014	1158	732	1021	lcl|ORF9_Mya_contig00670:1891:32 [Mya truncata11]	619	166	446	314	45.4	23.25	1.00 E-06	46.6
CGI_10028014	1158	920	1149	lcl|ORF2_Mya_contig05387:841:104 [Mya truncata50]	245	2	224	237	91.02	22.36	2.00 E-10	56.6
CGI_10028014	1158	996	1141	pfu_aug1.0_954.1_58317.t1[P fucata70]	160	13	157	148	90.63	27.7	8.00 E-10	53.9
CGI_10028014	1158	633	725	gi|906541694|gb|AKS48142.1| collagen-like protein-2 [Mytilus coruscus20]	453	341	440	100	22.08	32	1.00 E-08	52.8

CGI_10028014	1158	919	1032	pfu_aug1.0_21286.1_69411.t1[P fucata64]	152	32	141	117	72.37	29.91	4.00 E-08	48.5
CGI_10028014	1158	788	893	lcl|ORF12_Mya_contig00629:1875:85 [Mya truncata12]	596	66	184	120	19.97	28.33	5.00 E-08	50.8
CGI_10028014	1158	848	1102	lcl|ORF12_Mya_contig00629:1875:85 [Mya truncata12]	596	203	506	324	51.01	23.46	8.00 E-07	47
CGI_10028014	1158	1032	1151	gi|374253727|sp|B3A0P4.1|USP26_LOTGI RecName: Full=Uncharacterized shell protein 26; AltName: Full=BMSP-like protein [Lgigantea36]	173	49	169	122	69.94	26.23	1.00 E-07	47.4
CGI_10028014	1158	649	765	pfu_aug1.0_12760.1_53855.t1[P fucata48]	400	30	140	117	27.75	24.79	2.00 E-07	48.9
CGI_10028014	1158	809	953	lcl|ORF8_Mya_contig01412:1442:3 [Mya truncata5]	480	140	279	153	29.17	27.45	3.00 E-07	48.1
CGI_10028014	1158	638	700	pfu_aug1.0_287428.1_50307.t1[P fucata41]	155	87	149	63	40.65	36.51	2.00 E-06	43.5
CGI_10028014	1158	908	1007	gi|906541789|gb|AKS48174.1| KSL-rich protein-1, partial [Mytilus coruscus52]	372	282	371	101	24.19	26.73	1.00 E-05	43.1
CGI_10003000	723	600	720	pfu_aug1.0_14219.1_03462.t1[P fucata9]	121	1	121	121	100	30.58	3.00 E-10	54.3
CGI_10003000	723	527	721	gi|906541745|gb|AKS48159.1| EP-protein-1 [Mytilus coruscus37]	236	68	236	195	71.61	27.18	8.00 E-10	53.9
CGI_10012348	5788	177	1378	gi|906541676|gb|AKS48136.1| heparan sulfate proteoglycan-like protein-1 [Mytilus coruscus14]	4278	2458	3537	1244	25.25	21.3	4.00 E-24	107
CGI_10012348	5788	85	760	gi|906541676|gb|AKS48136.1| heparan sulfate proteoglycan-like protein-1 [Mytilus coruscus14]	4278	2923	3536	699	14.35	22.75	6.00 E-23	103
CGI_10012348	5788	82	1061	gi|906541676|gb|AKS48136.1| heparan sulfate proteoglycan-like protein-1 [Mytilus coruscus14]	4278	2545	3451	1018	21.2	20.92	1.00 E-21	99.4
CGI_10012348	5788	55	654	gi|906541676|gb|AKS48136.1| heparan sulfate proteoglycan-like protein-1 [Mytilus coruscus14]	4278	2978	3537	625	13.09	23.36	4.00 E-20	94
CGI_10012348	5788	1106	2192	gi|906541676|gb|AKS48136.1| heparan sulfate proteoglycan-like protein-1 [Mytilus coruscus14]	4278	2470	3538	1171	24.99	22.54	2.00 E-19	92
CGI_10012348	5788	4052	5235	gi|906541676|gb|AKS48136.1| heparan sulfate proteoglycan-like protein-1 [Mytilus coruscus14]	4278	2459	3533	1261	25.13	23	5.00 E-17	84

CGI_10012348	5788	1187	2274	gi|906541676|gb|AKS48136.1| heparan sulfate proteoglycan-like protein-1 [Mytilus coruscus14]	4278	2458	3531	1176	25.11	21.51	3.00 E-16	80.9
CGI_10012348	5788	1604	2729	gi|906541676|gb|AKS48136.1| heparan sulfate proteoglycan-like protein-1 [Mytilus coruscus14]	4278	2580	3536	1176	22.37	19.98	3.00 E-15	78.2
CGI_10012348	5788	3530	4211	gi|906541676|gb|AKS48136.1| heparan sulfate proteoglycan-like protein-1 [Mytilus coruscus14]	4278	2846	3531	747	16.04	23.69	8.00 E-14	73.2
CGI_10012348	5788	3544	4403	gi|906541676|gb|AKS48136.1| heparan sulfate proteoglycan-like protein-1 [Mytilus coruscus14]	4278	2673	3531	941	20.08	20.72	4.00 E-10	60.8
CGI_10012348	5788	3754	4732	gi|906541676|gb|AKS48136.1| heparan sulfate proteoglycan-like protein-1 [Mytilus coruscus14]	4278	2595	3544	1063	22.21	22.58	5.00 E-10	60.5
CGI_10012348	5788	2078	3222	gi|906541676|gb|AKS48136.1| heparan sulfate proteoglycan-like protein-1 [Mytilus coruscus14]	4278	2509	3556	1220	24.5	20.57	6.00 E-10	60.1
CGI_10012348	5788	4558	5425	gi|906541676|gb|AKS48136.1| heparan sulfate proteoglycan-like protein-1 [Mytilus coruscus14]	4278	2746	3534	905	18.44	20.88	2.00 E-09	58.5
CGI_10012348	5788	3860	4912	gi|906541676|gb|AKS48136.1| heparan sulfate proteoglycan-like protein-1 [Mytilus coruscus14]	4278	2517	3535	1168	23.82	21.66	1.00 E-08	55.8
CGI_10012348	5788	2013	2857	gi|906541676|gb|AKS48136.1| heparan sulfate proteoglycan-like protein-1 [Mytilus coruscus14]	4278	2725	3459	872	17.18	21.1	5.00 E-08	53.9
CGI_10012348	5788	5109	5424	gi|906541676|gb|AKS48136.1| heparan sulfate proteoglycan-like protein-1 [Mytilus coruscus14]	4278	2500	2803	331	7.11	22.05	8.00 E-06	46.6
CGI_10012348	5788	5538	5783	lcl|ORF2_Mya_contig05387:841:104 [Mya truncata50]	245	15	228	256	87.35	25	1.00 E-11	62.4
CGI_10012348	5788	581	859	gi|906541688|gb|AKS48140.1| twitchin-like protein-1 [Mytilus coruscus18]	3229	2923	3196	294	8.49	24.15	5.00 E-11	63.9
CGI_10012348	5788	1676	1956	gi|906541688|gb|AKS48140.1| twitchin-like protein-1 [Mytilus coruscus18]	3229	2920	3201	301	8.73	23.92	1.00 E-10	62.4
CGI_10012348	5788	3812	4046	gi|906541688|gb|AKS48140.1| twitchin-like protein-1 [Mytilus coruscus18]	3229	2931	3198	271	8.3	21.03	2.00 E-07	52.4
CGI_10012348	5788	400	661	gi|906541688|gb|AKS48140.1| twitchin-like protein-1 [Mytilus coruscus18]	3229	2929	3205	292	8.58	22.95	1.00 E-06	49.7
CGI_10012348	5788	4671	4811	gi|906541688|gb|AKS48140.1| twitchin-like protein-1 [Mytilus coruscus18]	3229	3053	3198	149	4.52	27.52	3.00 E-06	48.1

CGI_10012348	5788	5538	5781	gi|906541679|gb|AKS48137.1| matrix protein-1 [Mytilus coruscus15]	635	414	632	251	34.49	25.5	3.00 E-10	60.5
CGI_10012348	5788	5407	5781	pfu_aug1.0_1843.1_37145.t1[P fucata52]	1923	1566	1920	398	18.46	22.11	4.00 E-09	57.8
CGI_10012348	5788	1583	1911	gi|906541691|gb|AKS48141.1| twitchin-like protein-2, partial [Mytilus coruscus19]	435	17	359	374	78.85	22.19	5.00 E-09	56.2
CGI_10012348	5788	392	764	gi|906541691|gb|AKS48141.1| twitchin-like protein-2, partial [Mytilus coruscus19]	435	15	412	421	91.49	20.67	1.00 E-07	51.6
CGI_10012348	5788	3632	3963	gi|906541691|gb|AKS48141.1| twitchin-like protein-2, partial [Mytilus coruscus19]	435	22	408	396	88.97	21.72	7.00 E-07	49.3
CGI_10012348	5788	1107	1470	gi|906541691|gb|AKS48141.1| twitchin-like protein-2, partial [Mytilus coruscus19]	435	23	407	412	88.51	24.03	4.00 E-06	47
CGI_10012348	5788	5462	5785	gi|906541739|gb|AKS48157.1| sushi-like protein [Mytilus coruscus35]	844	491	844	377	41.94	22.81	7.00 E-09	56.2
CGI_10012348	5788	5446	5555	ML5H8[Hasinina14] 44.7	110	9	110	115	92.73	26.09	3.00 E-06	
CGI_10017543	371	29	349	gi|353558795|sp|P86953.1|ELDP1_PINMA RecName: Full=EGF-like domain-containing protein 1; Flags: Precursor[Pmaxima14]	348	17	330	321	90.23	42.37	3.00 E-91	276
CGI_10017543	371	29	349	gi|391359264|sp|H2A0L2.1|ELDP1_PINMG RecName: Full=EGF-like domain containing protein 1; Flags: Precursor [Pinctada margaritifera8]	348	17	330	321	90.23	40.5	1.00 E-85	261
CGI_10017543	371	18	349	gi|391359265|sp|H2A0L3.1|ELDP2_PINMG RecName: Full=EGF-like domain containing protein 2; Flags: Precursor [Pinctada margaritifera9]	359	7	330	336	90.25	33.33	2.00 E-52	175
CGI_10017543	371	14	349	gi|353558796|sp|P86954.1|ELDP2_PINMA RecName: Full=EGF-like domain-containing protein 2; Flags: Precursor [Pmaxima15]	357	5	328	339	90.76	32.74	3.00 E-51	172
CGI_10017543	371	14	345	pfu_aug1.0_853.1_22356.t1[P fucata56]	363	5	327	336	88.98	32.44	1.00 E-48	165
CGI_10017543	371	17	360	gi|374110557|sp|B3A0R6.1|ELDP1_LOTGI RecName: Full=EGF-like domain-containing protein 1; AltName:	513	9	368	383	70.18	22.98	3.00 E-15	71.2

				Full=Uncharacterized shell protein 17; Short=LUSP-17; Flags: Precursor[Lgigantea14]								
CGI_10017543	371	31	294	gi|374110558|sp|B3A0S3.1|ELDP2_LOTGI RecName: Full=EGF-like domain-containing protein 2; AltName: Full=Uncharacterized shell protein 24; Short=LUSP-24; Flags: Precursor[Lgigantea15]	489	23	306	301	58.08	24.58	8.00 E-14	66.6
CGI_10023765	854	100	843	gi|906541769|gb|AKS48167.1| protease inhibitor-like protein-D2 [Mytilus coruscus45]	1205	457	1204	765	62.07	43.4	0	642
CGI_10023765	854	267	840	lcl|ORF18_Mya_contig00475:1794:70 [Mya truncata14]	574	4	572	579	99.13	48.53	0	557
CGI_10023765	854	126	838	lcl|ORF7_Mya_contig00348:2331:133 [Mya truncata15]	732	4	717	741	97.54	38.19	4.00 E-161	486
CGI_10016965	630	5	523	gi|824631368|gb|AKI87977.1| shell protein-6 [Mytilus coruscus6]	606	31	578	555	90.43	46.85	1.00 E-146	437
CGI_10016965	630	1	521	gi|391359279|sp|H2A0L8.1|FND2_PINMG RecName: Full=Fibronectin type III domain-containing protein 2; Flags: Precursor[Pinctada margaritifera11]	624	43	607	569	90.54	44.64	2.00 E-139	419
CGI_10016965	630	25	528	gi|391359278|sp|H2A0L7.1|FND1_PINMG RecName: Full=Fibronectin type III domain-containing protein 1; Flags: Precursor[Pinctada margaritifera10]	754	202	729	538	70.03	38.66	1.00 E-106	337
CGI_10016965	630	102	441	pfu_aug1.0_11437.1_03076.t1[P fucata47]	392	8	390	386	97.7	38.34	2.00 E-77	249
CGI_10016965	630	296	469	pfu_aug1.0_11437.1_03076.t1[P fucata47]	392	33	220	191	47.96	23.56	7.00 E-08	48.9
CGI_10016965	630	250	391	pfu_aug1.0_155246.1_57288.t1[P fucata50]	144	2	144	143	99.31	51.75	8.00 E-45	152
CGI_10016965	630	78	242	pfu_aug1.0_13143.1_39453.t1[P fucata49]	306	110	303	195	63.4	45.64	9.00 E-38	137
CGI_10016965	630	458	529	pfu_aug1.0_154829.1_06667.t1[P fucata60]	127	3	74	72	56.69	62.5	3.00 E-24	94.7

CGI_10016965	630	138	246	pfu_aug1.0_9430.1_38910.t1[P fucata59]	165	30	162	134	80.61	32.09	2.00 E-14	67
CGI_10016965	630	200	288	pfu_aug1.0_9430.1_38910.t1[P fucata59]	165	22	107	92	52.12	35.87	2.00 E-08	48.1
CGI_10016397	4990	189	590	gi|906541766|gb|AKS48166.1| tyrosinase-like protein-1, partial [Mytilus coruscus44]	578	1	388	421	67.13	33.49	4.00 E-51	189
CGI_10016397	4990	840	989	gi|906541766|gb|AKS48166.1| tyrosinase-like protein-1, partial [Mytilus coruscus44]	578	237	391	157	26.82	35.03	2.00 E-23	102
CGI_10016397	4990	288	582	lcl|ORF1_Mya_contig00902:31:1749 [Mya truncata7]	573	66	372	315	53.58	37.78	1.00 E-47	179
CGI_10016397	4990	833	980	lcl|ORF1_Mya_contig00902:31:1749 [Mya truncata7]	573	218	374	159	27.4	36.48	2.00 E-19	89.7
CGI_10016397	4990	714	818	lcl|ORF1_Mya_contig00902:31:1749 [Mya truncata7]	573	448	569	123	21.29	30.08	1.00 E-10	62
CGI_10016397	4990	1109	1220	lcl|ORF1_Mya_contig00902:31:1749 [Mya truncata7]	573	436	568	133	23.21	30.08	2.00 E-10	61.2
CGI_10016397	4990	189	592	gi|391359338|sp|H2A0L1.1|TYRO2_PINMG RecName: Full=Tyrosinase-like protein 2; AltName: Full=Tyrosinase 2; Flags: Precursor[Pinctada margaritifera27]	456	4	411	446	89.47	29.6	3.00 E-36	142
CGI_10016397	4990	863	995	gi|391359338|sp|H2A0L1.1|TYRO2_PINMG RecName: Full=Tyrosinase-like protein 2; AltName: Full=Tyrosinase 2; Flags: Precursor[Pinctada margaritifera27]	456	290	416	134	27.85	38.81	7.00 E-16	77.8
CGI_10016397	4990	219	581	gi|391359337|sp|H2A0L0.1|TYRO1_PINMG RecName: Full=Tyrosinase-like protein 1; AltName: Full=Tyrosinase 1; Flags: Precursor[Pinctada margaritifera26]	492	35	390	383	72.36	32.64	9.00 E-36	140
CGI_10016397	4990	862	988	gi|391359337|sp|H2A0L0.1|TYRO1_PINMG RecName: Full=Tyrosinase-like protein 1; AltName: Full=Tyrosinase 1; Flags: Precursor[Pinctada margaritifera26]	492	283	407	134	25.41	38.06	7.00 E-16	78.2

CGI_10016397	4990	262	592	gi|353558891|sp|P86952.1|TYRO_PINMA RecName: Full=Tyrosinase-like protein; AltName: Full=Tyrosinase-2; Flags: Precursor[Pmaxima17]	456	71	411	368	74.78	30.98	4.00 E-35	138
CGI_10016397	4990	863	995	gi|353558891|sp|P86952.1|TYRO_PINMA RecName: Full=Tyrosinase-like protein; AltName: Full=Tyrosinase-2; Flags: Precursor[Pmaxima17]	456	290	416	134	27.85	38.06	8.00 E-16	77.8
CGI_10016397	4990	243	592	pfu_aug1.0_16905.1_25558.t1[P fucata18]	442	33	390	390	81	29.49	2.00 E-33	133
CGI_10016397	4990	849	1009	pfu_aug1.0_16905.1_25558.t1[P fucata18]	442	254	418	171	37.33	32.75	3.00 E-15	75.9
CGI_10016397	4990	339	581	pfu_aug1.0_3212.1_37533.t1[P fucata15]	430	36	295	268	60.47	32.09	1.00 E-29	120
CGI_10016397	4990	859	987	pfu_aug1.0_3212.1_37533.t1[P fucata15]	430	181	311	138	30.47	34.78	8.00 E-15	74.3
CGI_10016397	4990	337	592	pfu_aug1.0_12145.1_17832.t1[P fucata16]	315	1	277	294	87.94	31.29	3.00 E-29	118
CGI_10016397	4990	849	1009	pfu_aug1.0_12145.1_17832.t1[P fucata16]	315	141	305	173	52.38	32.37	1.00 E-15	75.5
CGI_10016397	4990	346	581	pfu_aug1.0_10251.1_39018.t1[P fucata8]	390	2	254	260	64.87	31.92	3.00 E-28	116
CGI_10016397	4990	859	987	pfu_aug1.0_10251.1_39018.t1[P fucata8]	390	140	270	138	33.59	34.78	6.00 E-15	74.3
CGI_10016397	4990	370	581	pfu_aug1.0_21093.1_62062.t1[P fucata17]	477	151	378	233	47.8	31.76	2.00 E-24	104
CGI_10016397	4990	859	977	pfu_aug1.0_21093.1_62062.t1[P fucata17]	477	263	378	123	24.32	37.4	2.00 E-13	70.5
CGI_10016397	4990	392	639	A1256_Magellania1208_r10023620[Mvenosa34] 103	376	3	274	279	72.34	28.32	3.00 E-24	
CGI_10016397	4990	833	1035	A1256_Magellania1208_r10023620[Mvenosa34] 75.9	376	64	274	222	56.12	27.48	2.00 E-15	
CGI_10020612	549	35	108	pfu_aug1.0_75094.1_56521.t1[P fucata43]	145	1	77	78	53.1	29.49	3.00 E-06	41.6

CGI_10023851	253	89	242	gi|374110750|sp|B3A0R0.1|PPI_LOTGI RecName: Full=Putative peptidyl-prolyl cis-trans isomerase; Short=PPIase; Flags: Precursor[Lgigantea27]	206	40	189	154	72.82	57.14	3.00 E-58	182
CGI_10012352	1115	721	1115	gi|906541739|gb|AKS48157.1| sushi-like protein [Mytilus coruscus35]	844	489	840	404	41.71	25.99	2.00 E-29	120
CGI_10012352	1115	15	315	gi|906541739|gb|AKS48157.1| sushi-like protein [Mytilus coruscus35]	844	545	818	312	32.46	23.4	2.00 E-16	78.6
CGI_10012352	1115	869	1113	lcl|ORF2_Mya_contig05387:841:104 [Mya truncata50]	245	14	224	246	86.12	24.8	2.00 E-18	81.3
CGI_10012352	1115	107	341	lcl|ORF2_Mya_contig05387:841:104 [Mya truncata50]	245	14	217	241	83.27	23.65	1.00 E-13	66.2
CGI_10012352	1115	720	1115	pfu_aug1.0_1843.1_37145.t1[P fucata52]	1923	1571	1920	405	18.2	22.47	4.00 E-17	80.9
CGI_10012352	1115	9	341	pfu_aug1.0_1843.1_37145.t1[P fucata52]	1923	1620	1911	344	15.18	25	3.00 E-15	75.1
CGI_10012352	1115	721	1115	gi|906541679|gb|AKS48137.1| matrix protein-1 [Mytilus coruscus15]	635	271	632	408	57.01	23.77	3.00 E-16	77.4
CGI_10012352	1115	721	965	gi|391359324|sp|H2A0N4.1|PIF_PINMG RecName: Full=Protein PIF; Contains: RecName: Full=Protein Pif97; Contains: RecName: Full=Protein Pif80; AltName: Full=Aragonite-binding protein;Flags: Precursor [Pinctada margaritifera20]	1014	275	529	266	25.15	26.32	1.00 E-12	65.9
CGI_10012352	1115	12	205	gi|391359324|sp|H2A0N4.1|PIF_PINMG RecName: Full=Protein PIF; Contains: RecName: Full=Protein Pif97; Contains: RecName: Full=Protein Pif80; AltName: Full=Aragonite-binding protein;Flags: Precursor [Pinctada margaritifera20]	1014	332	537	218	20.32	24.31	9.00 E-08	50.1
CGI_10012352	1115	21	348	lcl|ORF9_Mya_contig00670:1891:32 [Mya truncata11]	619	285	580	340	47.82	25	4.00 E-12	63.9
CGI_10012352	1115	758	1113	lcl|ORF9_Mya_contig00670:1891:32 [Mya truncata11]	619	254	590	380	54.44	22.37	6.00 E-12	63.5
CGI_10012352	1115	683	820	lcl|ORF9_Mya_contig00670:1891:32 [Mya truncata11]	619	47	189	147	23.1	27.21	2.00 E-07	49.3

CGI_10012352	1115	723	823	gi|175684/239574|PA_LOTGI RecName:3 peritrophin-A/CBM_14 AltName: Full=Uncharacterized shell protein 20; Short=LUSP-20[Lgigantea5]	716	403	501	101	13.83	27.72	2.00 E-10	58.9
CGI_10012352	1115	723	826	lcl|ORF1_Mya_contig00894:20:1558 [Mya truncata8]	512	9	116	110	21.09	32.73	6.00 E-09	53.5
CGI_10012352	1115	858	1115	gi|391359317|sp|H2A0M0.1|NRP_PINMG RecName: Full=Asparagine-rich protein; AltName: Full=Prism uncharacterized shell protein 1; Short=PUSP1; Flags: Precursor[Pinctada margaritifera19]	686	453	679	263	33.09	19.77	4.00 E-08	51.2
CGI_10012352	1115	76	293	gi|391359317|sp|H2A0M0.1|NRP_PINMG RecName: Full=Asparagine-rich protein; AltName: Full=Prism uncharacterized shell protein 1; Short=PUSP1; Flags: Precursor[Pinctada margaritifera19]	686	425	633	235	30.47	25.53	5.00 E-08	50.8
CGI_10012352	1115	743	844	lcl|ORF1_Mya_contig01082:16:1497 [Mya truncata6]	493	1	108	109	21.91	29.36	2.00 E-07	48.9
CGI_10012352	1115	21	317	lcl|ORF12_Mya_contig00629:1875:85 [Mya truncata12]	596	265	528	306	44.3	22.22	7.00 E-07	47
CGI_10012352	1115	756	1113	lcl|ORF12_Mya_contig00629:1875:85 [Mya truncata12]	596	236	567	379	55.7	21.37	8.00 E-07	47
CGI_10012352	1115	755	817	lcl|ORF12_Mya_contig00629:1875:85 [Mya truncata12]	596	173	234	63	10.4	33.33	9.00 E-06	43.5
CGI_10012352	1115	946	1113	lcl|ORF6_Mya_contig01866:900:1382 [Mya truncata3]	161	9	161	174	95.03	25.29	7.00 E-07	44.7
CGI_10012352	1115	1045	1115	gi|374253727|sp|B3A0P4.1|USP26_LOTGI RecName: Full=Uncharacterized shell protein 26; AltName: Full=BMSP-like protein [Lgigantea36]	173	108	169	71	35.84	29.58	9.00 E-06	41.6
CGI_10020756	739	686	736	gi|391359293|sp|H2A0N9.1|KCP4_PINMG RecName: Full=BPTI/Kunitz domain-containing protein 4; AltName: Full=Nacre serine protease inhibitor 3; Short=NSPI3; Flags: Precursor [Pinctada margaritifera16]	198	146	196	51	25.76	52.94	5.00 E-15	69.7
CGI_10020756	739	695	736	gi|906541736|gb|AKS48156.1| protease inhibitor-like protein-C [Mytilus coruscus34]	175	132	173	42	24	52.38	8.00 E-11	56.2

CGI_10020756	739	686	736	P0012N13_463[Hasinina3] 52.4	126	70	120	51	40.48	39.22	1.00 E-09	
CGI_10020756	739	679	738	P0012N13_463[Hasinina3] 50.1	126	7	66	60	47.62	33.33	6.00 E-09	
CGI_10020756	739	700	736	gi|391359294|sp|H2A0P0.1|KCP5_PINMG RecName: Full=BPTI/Kunitz domain-containing protein 5; AltName: Full=Nacre serine protease inhibitor 5; Flags: Precursor[Pinctada margaritifera17]	182	105	141	37	20.33	51.35	1.00 E-08	49.7
CGI_10020756	739	686	736	gi|391359294|sp|H2A0P0.1|KCP5_PINMG RecName: Full=BPTI/Kunitz domain-containing protein 5; AltName: Full=Nacre serine protease inhibitor 5; Flags: Precursor[Pinctada margaritifera17]	182	29	81	53	29.12	37.74	6.00 E-07	44.7
CGI_10020756	739	693	736	gi|391359290|sp|H2A0M2.1|KCP1_PINMG RecName: Full=BPTI/Kunitz domain-containing protein 1; AltName: Full=Prism serine protease inhibitor 1; Short=PSPI1; Flags: Precursor[Pinctadamargaritifera13]	138	79	122	44	31.88	40.91	3.00 E-08	47.8
CGI_10020756	739	684	736	gi|353558671|sp|P86959.1|KCP1_PINMA RecName: Full=BPTI/Kunitz domain-containing protein 1; AltName: Full=Prism serine protease inhibitor 1; Short=PSPI1; Flags: Precursor[Pmaxima9]	138	70	122	53	38.41	33.96	4.00 E-08	47.4
CGI_10020756	739	693	738	gi|391359291|sp|H2A0N1.1|KCP2_PINMG RecName: Full=BPTI/Kunitz domain-containing protein 2; AltName: Full=Prism serine protease inhibitor 2; Short=PISP2; Flags: Precursor [Pinctadamargaritifera14]	141	38	81	46	31.21	45.65	3.00 E-07	44.7
CGI_10020756	739	693	736	gi|391359291|sp|H2A0N1.1|KCP2_PINMG RecName: Full=BPTI/Kunitz domain-containing protein 2; AltName: Full=Prism serine protease inhibitor 2; Short=PISP2; Flags: Precursor [Pinctadamargaritifera14]	141	92	136	45	31.91	42.22	2.00 E-06	42.4
CGI_10020756	739	701	736	gi|391359292|sp|H2A0N5.1|KCP3_PINMG RecName: Full=BPTI/Kunitz domain-containing protein 3;	157	60	95	36	22.93	50	3.00 E-06	42

				AltName: Full=Nacre serine protease inhibitor 2; Flags: Precursor [Pinctadamargaritifera15]								
CGI_10020756	739	701	736	gi|353558673|sp|P86964.1|KCP3_PINMA RecName: Full=BPTI/Kunitz domain-containing protein 3; AltName: Full=Nacre serine protease inhibitor 2; Short=NSPI2; Flags: Precursor[Pmaxima11]	157	60	95	36	22.93	50	3.00 E-06	42
CGI_10007857	1002	13	845	gi|391359282|sp|H2A0L6.1|HEX_PINMG RecName: Full=Putative beta-hexosaminidase; AltName: Full=Beta-N-acetylhexosaminidase;AltName: Full=Chitobiase; AltName: Full=N-acetyl-beta-glucosaminidase;Flags: Precursor [Pinctada margaritifera12]	1135	30	846	870	71.98	32.53	7.00 E-116	381
CGI_10007857	1002	13	845	gi|353558649|sp|P86956.1|HEX_PINMA RecName: Full=Putative beta-hexosaminidase; AltName: Full=Beta-N-acetylhexosaminidase;AltName: Full=Chitobiase; AltName: Full=N-acetyl-beta-glucosaminidase;Flags: Precursor [Pmaxima8]	1130	30	845	865	72.21	32.25	2.00 E-110	366
CGI_10014170	543	5	382	gi|906541754|gb|AKS48162.1| nacrein-like protein-2 [Mytilus coruscus40]	373	7	361	386	95.17	47.67	5.00 E-108	326
CGI_10014170	543	5	382	gi|824631529|gb|AKI87981.1| nacrein-like protein [Mytilus coruscus10]	372	7	360	390	95.16	44.62	2.00 E-97	298
CGI_10014170	543	41	215	gi|74826594|sp|Q9NL38.1|MA66_PINMA RecName: Full=N66 matrix protein; Flags: Precursor[Pmaxima1]	568	54	218	185	29.05	30.81	9.00 E-18	80.5
CGI_10014170	543	313	378	gi|74826594|sp|Q9NL38.1|MA66_PINMA RecName: Full=N66 matrix protein; Flags: Precursor[Pmaxima1]	568	484	552	71	12.15	45.07	5.00 E-10	55.8
CGI_10014170	543	309	378	pfu_aug1.0_8238.1_17260.t1[P fucata3]	269	182	254	75	27.14	46.67	7.00 E-13	63.2
CGI_10014170	543	40	212	lcl|ORF4_Mya_contig00764:66:1418 [Mya truncata22]	450	41	207	182	37.11	30.22	9.00 E-13	64.3
CGI_10014170	543	325	364	lcl|ORF4_Mya_contig00764:66:1418 [Mya truncata22]	450	254	293	40	8.89	50	1.00 E-08	51.6

CGI_10014170	543	39	353	gi|374110482|sp|B3A0P2.1|CAH1_LOTGI RecName: Full=Putative carbonic anhydrase 1; AltName: Full=Putative carbonate dehydratase 1; Flags: Precursor [Lgigantea7]	395	37	325	318	73.16	22.96	1.00 E-12	63.9
CGI_10014170	543	46	211	gi|906541712|gb|AKS48148.1| carbonic anhydrase-like protein, partial [Mytilus coruscus26]	321	26	169	172	44.86	30.81	2.00 E-11	59.7
CGI_10014170	543	318	368	gi|906541712|gb|AKS48148.1| carbonic anhydrase-like protein, partial [Mytilus coruscus26]	321	205	258	54	16.82	40.74	7.00 E-07	45.1
CGI_10014170	543	43	215	lcl|ORF7_contig_25891:609:3695 [Cnemoralis46]	1028	39	193	187	15.08	29.41	1.00 E-10	58.2
CGI_10014170	543	316	354	lcl|ORF7_contig_25891:609:3695 [Cnemoralis46]	1028	222	261	40	3.89	52.5	4.00 E-06	43.5
CGI_10014170	543	313	355	gi|374110483|sp|B3A0Q6.1|CAH2_LOTGI RecName: Full=Putative carbonic anhydrase 2[Lgigantea8]	448	142	184	43	9.6	44.19	2.00 E-07	47
CGI_10026603	1290	30	541	lcl|ORF17_Mya_contig00108:2971:632 [Mya truncata18]	779	9	514	527	64.96	52.18	3.00 E-175	537
CGI_10026603	1290	623	990	lcl|ORF17_Mya_contig00108:2971:632 [Mya truncata18]	779	24	396	375	47.88	60	1.00 E-151	475
CGI_10026603	1290	998	1220	lcl|ORF17_Mya_contig00108:2971:632 [Mya truncata18]	779	302	514	231	27.34	45.45	5.00 E-50	185
CGI_10026603	1290	475	526	lcl|ORF17_Mya_contig00108:2971:632 [Mya truncata18]	779	720	772	53	6.8	35.85	9.00 E-07	47
CGI_10026603	1290	1154	1205	lcl|ORF17_Mya_contig00108:2971:632 [Mya truncata18]	779	720	772	53	6.8	35.85	9.00 E-07	47
CGI_10026603	1290	28	520	gi|906541763|gb|AKS48165.1| chitinase-like protein-1, partial [Mytilus coruscus43]	446	6	445	503	98.65	38.57	1.00 E-101	328
CGI_10026603	1290	613	993	gi|906541763|gb|AKS48165.1| chitinase-like protein-1, partial [Mytilus coruscus43]	446	16	362	392	77.8	41.84	8.00 E-91	297
CGI_10026603	1290	983	1199	gi|906541763|gb|AKS48165.1| chitinase-like protein-1, partial [Mytilus coruscus43]	446	250	445	218	43.95	32.11	8.00 E-24	100

CGI_10026603	1290	33	408	gi|391738044|sp|H2A0L4.1|CHI1_PINMG RecName: Full=Putative chitinase 1; AltName: Full=Chitinase-like protein 1; Short=Clp1;Flags: Precursor [Pinctada margaritifera43]	468	7	374	387	78.63	35.4	9.00 E-76	255
CGI_10026603	1290	621	1003	gi|391738044|sp|H2A0L4.1|CHI1_PINMG RecName: Full=Putative chitinase 1; AltName: Full=Chitinase-like protein 1; Short=Clp1;Flags: Precursor [Pinctada margaritifera43]	468	19	391	393	79.7	34.1	2.00 E-71	243
CGI_10026603	1290	1011	1087	gi|391738044|sp|H2A0L4.1|CHI1_PINMG RecName: Full=Putative chitinase 1; AltName: Full=Chitinase-like protein 1; Short=Clp1;Flags: Precursor [Pinctada margaritifera43]	468	299	374	77	16.24	46.75	3.00 E-20	89.7
CGI_10026603	1290	30	408	pfu_aug1.0_10761.1_31980.t1[P fucata20]	503	36	410	392	74.55	33.16	7.00 E-69	237
CGI_10026603	1290	624	991	pfu_aug1.0_10761.1_31980.t1[P fucata20]	503	57	416	377	71.57	32.89	5.00 E-65	226
CGI_10026603	1290	1009	1087	pfu_aug1.0_10761.1_31980.t1[P fucata20]	503	333	410	79	15.51	39.24	2.00 E-17	80.9
CGI_10026603	1290	33	408	gi|391738045|sp|H2A0L5.1|CHI2_PINMG RecName: Full=Putative chitinase 2; AltName: Full=Chitinase-like protein 3; Short=Clp3;Flags: Precursor[Pinctada margaritifera44]	466	7	373	385	78.76	33.25	1.00 E-68	235
CGI_10026603	1290	624	985	gi|391738045|sp|H2A0L5.1|CHI2_PINMG RecName: Full=Putative chitinase 2; AltName: Full=Chitinase-like protein 3; Short=Clp3;Flags: Precursor[Pinctada margaritifera44]	466	20	373	371	75.97	32.08	5.00 E-64	222
CGI_10026603	1290	1009	1087	gi|391738045|sp|H2A0L5.1|CHI2_PINMG RecName: Full=Putative chitinase 2; AltName: Full=Chitinase-like protein 3; Short=Clp3;Flags: Precursor[Pinctada margaritifera44]	466	296	373	79	16.74	37.97	1.00 E-16	78.6
CGI_10026603	1290	33	408	gi|353558824|sp|P86955.1|CHI_PINMA RecName: Full=Putative chitinase; AltName: Full=Chitinase-like protein 3; Short=Clp3;Flags: Precursor[Pmaxima16]	466	7	373	385	78.76	33.25	2.00 E-68	234

CGI_10026603	1290	612	985	gi|353558824|sp|P86955.1|CHI_PINMA RecName: Full=Putative chitinase; AltName: Full=Chitinase-like protein 3; Short=Clp3;Flags: Precursor[Pmaxima16]	466	4	373	387	79.4	32.3	8.00 E-65	224
CGI_10026603	1290	1009	1087	gi|353558824|sp|P86955.1|CHI_PINMA RecName: Full=Putative chitinase; AltName: Full=Chitinase-like protein 3; Short=Clp3;Flags: Precursor[Pmaxima16]	466	296	373	79	16.74	37.97	1.00 E-16	78.2
CGI_10026603	1290	202	408	pfu_aug1.0_14887.1_32490.t1[P fucata39]	300	13	206	207	64.67	34.78	2.00 E-33	127
CGI_10026603	1290	779	1003	pfu_aug1.0_14887.1_32490.t1[P fucata39]	300	13	223	226	70.33	33.19	4.00 E-31	121
CGI_10026603	1290	1011	1087	pfu_aug1.0_14887.1_32490.t1[P fucata39]	300	131	206	77	25.33	48.05	2.00 E-20	88.6
CGI_10026603	1290	47	200	pfu_aug1.0_13989.1_32380.t1[P fucata19]	396	1	151	157	38.13	36.31	9.00 E-33	127
CGI_10026603	1290	624	772	pfu_aug1.0_13989.1_32380.t1[P fucata19]	396	1	147	152	37.12	36.18	1.00 E-32	127
CGI_10026603	1290	332	408	pfu_aug1.0_13989.1_32380.t1[P fucata19]	396	227	302	77	19.19	48.05	4.00 E-21	91.7
CGI_10026603	1290	1011	1087	pfu_aug1.0_13989.1_32380.t1[P fucata19]	396	227	302	77	19.19	48.05	4.00 E-21	91.7
CGI_10026603	1290	909	985	pfu_aug1.0_13989.1_32380.t1[P fucata19]	396	227	302	77	19.19	44.16	2.00 E-19	86.3
CGI_10026603	1290	384	598	lcl|ORF5_contig_248122:627:1 [Cnemoralis59]	209	1	178	216	85.17	28.7	6.00 E-20	85.9
CGI_10026603	1290	1063	1277	lcl|ORF5_contig_248122:627:1 [Cnemoralis59]	209	1	178	216	85.17	28.7	6.00 E-20	85.9
CGI_10012353	558	78	507	gi|906541739|gb|AKS48157.1| sushi-like protein [Mytilus coruscus35]	844	489	841	437	41.82	23.11	3.00 E-17	79.3
CGI_10012353	558	138	484	lcl|ORF9_Mya_contig00670:1891:32 [Mya truncata11]	619	216	544	376	53.15	22.61	3.00 E-11	59.7
CGI_10012353	558	79	448	lcl|ORF12_Mya_contig00629:1875:85 [Mya truncata12]	596	202	505	378	51.01	22.75	8.00 E-11	58.5

CGI_10012353	558	81	186	gi|175684/239574|PA_LOTGI RecName:3 peritrophin-A/CBM_14 AltName: Full=Uncharacterized shell protein 20; Short=LUSP-20[Lgigantea5]	716	404	511	116	15.08	24.14	4.00 E-10	56.6
CGI_10012353	558	76	187	gi|391359324|sp|H2A0N4.1|PIF_PINMG RecName: Full=Protein PIF; Contains: RecName: Full=Protein Pif97; Contains: RecName: Full=Protein Pif80; AltName: Full=Aragonite-binding protein;Flags: Precursor [Pinctada margaritifera20]	1014	273	376	112	10.26	31.25	5.00 E-10	56.2
CGI_10012353	558	78	447	gi|906541679|gb|AKS48137.1| matrix protein-1 [Mytilus coruscus15]	635	271	579	385	48.66	21.82	5.00 E-09	52.8
CGI_10012353	558	141	490	lcl|ORF1_Mya_contig01082:16:1497 [Mya truncata6]	493	114	454	370	69.17	21.35	2.00 E-07	47.8
CGI_10012353	558	216	463	lcl|ORF8_Mya_contig01412:1442:3 [Mya truncata5]	480	213	454	266	50.42	22.93	2.00 E-07	47.8
CGI_10012353	558	78	508	pfu_aug1.0_1843.1_37145.t1[P fucata52]	1923	1572	1922	437	18.25	18.54	6.00 E-06	43.1
CGI_10021733	849	431	817	A1256_Magellania1208_f20107906[Mvenosa18] 303	629	1	400	416	63.59	42.31	6.00 E-93	
CGI_10021733	849	198	775	gi|391359325|sp|H2A0M7.1|PLSP_PINMG RecName: Full=Peroxidase-like protein; Flags: Precursor [Pinctada margaritifera21]	793	160	747	612	74.15	32.84	7.00 E-88	293
CGI_10021733	849	206	512	A1256_Magellania1208_r10050836[Mvenosa35] 234	320	1	318	325	99.38	43.08	3.00 E-71	
CGI_10021733	849	464	774	gi|374110746|sp|B3A0Q8.1|PLSP3_LOTGI RecName: Full=Peroxidase-like protein 3[Lgigantea26]	294	2	287	313	97.28	31.95	7.00 E-46	162
CGI_10021733	849	520	781	gi|374110745|sp|B3A0P3.1|PLSP2_LOTGI RecName: Full=Peroxidase-like protein 2[Lgigantea25]	884	285	529	265	27.71	31.32	9.00 E-32	127
CGI_10021733	849	204	267	gi|374110745|sp|B3A0P3.1|PLSP2_LOTGI RecName: Full=Peroxidase-like protein 2[Lgigantea25]	884	11	83	73	8.26	42.47	6.00 E-08	50.4
CGI_10028414	734	567	652	lcl|ORF4_Mya_contig07764:573:280 [Mya truncata43]	97	14	89	86	78.35	44.19	8.00 E-09	49.3
CGI_10028414	734	219	304	lcl|ORF4_Mya_contig07764:573:280 [Mya truncata43]	97	14	89	86	78.35	43.02	5.00 E-08	47

CGI_10028414	734	485	573	lcl|ORF4_Mya_contig07764:573:280 [Mya truncata43]	97	9	92	90	86.6	38.89	9.00 E-07	43.1
CGI_10007021	782	585	674	lcl|ORF4_Mya_contig07764:573:280 [Mya truncata43]	97	9	92	91	86.6	40.66	2.00 E-08	48.1
CGI_10007021	782	679	754	lcl|ORF4_Mya_contig07764:573:280 [Mya truncata43]	97	22	91	76	72.16	43.42	3.00 E-08	47.8
CGI_10007021	782	506	595	lcl|ORF4_Mya_contig07764:573:280 [Mya truncata43]	97	12	94	91	85.57	36.26	4.00 E-06	41.2
CGI_10016430	143	20	104	pfu_aug1.0_75094.1_56521.t1[P fucata43]	145	1	84	85	57.93	55.29	9.00 E-29	100
CGI_10016430	143	20	105	gi|353558894|sp|P86958.1|USP1_PINMA RecName: Full=Uncharacterized shell protein 1; AltName: Full=Prism uncharacterized shell protein 2; Short=PUSP2; Flags: Precursor[Pmaxima18]	158	1	85	86	53.8	47.67	1.00 E-23	87.4
CGI_10016430	143	20	105	gi|391359262|sp|H2A0M1.1|DRP_PINMG RecName: Full=Aspartate-rich protein; AltName: Full=Prism uncharacterized shell protein 2; Short=PUSP2; Flags: Precursor [Pinctada margaritifera7]	152	1	85	86	55.92	48.84	1.00 E-23	87
CGI_10010359	550	301	539	gi|391359317|sp|H2A0M0.1|NRP_PINMG RecName: Full=Asparagine-rich protein; AltName: Full=Prism uncharacterized shell protein 1; Short=PUSP1; Flags: Precursor[Pinctada margaritifera19]	686	450	683	246	34.11	29.67	1.00 E-32	127
CGI_10010359	550	318	536	gi|906541739|gb|AKS48157.1| sushi-like protein [Mytilus coruscus35]	844	630	841	222	25.12	31.08	1.00 E-30	121
CGI_10010359	550	307	534	lcl|ORF2_Mya_contig05387:841:104 [Mya truncata50]	245	2	225	236	91.43	28.39	2.00 E-22	91.7
CGI_10010359	550	313	536	gi|906541679|gb|AKS48137.1| matrix protein-1 [Mytilus coruscus15]	635	408	633	228	35.59	28.07	2.00 E-21	92
CGI_10010359	550	303	537	pfu_aug1.0_1843.1_37145.t1[P fucata52]	1923	1695	1922	247	11.86	28.74	7.00 E-20	88.2
CGI_10010359	550	421	535	gi|374253727|sp|B3A0P4.1|USP26_LOTGI RecName: Full=Uncharacterized shell protein 26; AltName: Full=BMSP-like protein [Lgigantea36]	173	57	169	115	65.32	29.57	2.00 E-12	60.8

CGI_10010359	550	367	526	pfu_aug1.0_954.1_58317.t1[P fucata70]	160	2	159	165	98.75	24.85	1.00 E-09	52
CGI_10010359	550	292	413	pfu_aug1.0_13237.1_61173.t1[P fucata29]	177	38	165	128	72.32	28.91	1.00 E-08	48.9
CGI_10010359	550	257	390	gi|906541789|gb|AKS48174.1| KSL-rich protein-1, partial [Mytilus coruscus52]	372	256	372	137	31.45	26.28	2.00 E-08	50.1
CGI_10010359	550	315	414	gi|391359324|sp|H2A0N4.1|PIF_PINMG RecName: Full=Protein PIF; Contains: RecName: Full=Protein Pif97; Contains: RecName: Full=Protein Pif80; AltName: Full=Aragonite-binding protein;Flags: Precursor [Pinctada margaritifera20]	1014	433	538	106	10.45	32.08	1.00 E-07	48.1
CGI_10010359	550	377	530	gi|175684/239574|PA_LOTGI RecName:3 peritrophin-A/CBM_14 AltName: Full=Uncharacterized shell protein 20; Short=LUSP-20[Lgigantea5]	716	559	709	155	21.09	25.16	8.00 E-07	45.4
CGI_10014161	277	39	235	A1256_Magellania1208_r30126366[Mvenosa63] 97.4	348	150	347	207	56.9	33.33	3.00 E-25	
CGI_10014161	277	230	274	A1256_Magellania1208_r20126359[Mvenosa53] 41.6	276	1	46	46	16.67	43.48	3.00 E-06	
CGI_10026600	475	9	474	gi|391738044|sp|H2A0L4.1|CHI1_PINMG RecName: Full=Putative chitinase 1; AltName: Full=Chitinase-like protein 1; Short=Clp1;Flags: Precursor [Pinctada margaritifera43]	468	11	467	466	97.65	52.58	9.00 E-176	501
CGI_10026600	475	10	474	gi|353558824|sp|P86955.1|CHI_PINMA RecName: Full=Putative chitinase; AltName: Full=Chitinase-like protein 3; Short=Clp3;Flags: Precursor[Pmaxima16]	466	3	465	471	99.36	43.31	4.00 E-148	430
CGI_10026600	475	20	474	gi|391738045|sp|H2A0L5.1|CHI2_PINMG RecName: Full=Putative chitinase 2; AltName: Full=Chitinase-like protein 3; Short=Clp3;Flags: Precursor[Pinctada margaritifera44]	466	19	465	455	95.92	44.18	5.00 E-147	427
CGI_10026600	475	5	474	pfu_aug1.0_10761.1_31980.t1[P fucata20]	503	41	502	471	91.85	42.46	2.00 E-142	417

CGI_10026600	475	168	474	pfu_aug1.0_14887.1_32490.t1[P fucata39]	300	1	299	307	99.67	50.49	4.00 E-104	311
CGI_10026600	475	1	383	lcl|ORF17_Mya_contig00108:2971:632 [Mya truncata18]	779	5	395	395	50.19	32.41	9.00 E-64	217
CGI_10026600	475	21	188	pfu_aug1.0_13989.1_32380.t1[P fucata19]	396	1	168	168	42.42	53.57	8.00 E-62	204
CGI_10026600	475	304	474	pfu_aug1.0_13989.1_32380.t1[P fucata19]	396	227	395	171	42.68	53.8	2.00 E-56	190
CGI_10026600	475	19	383	gi|906541763|gb|AKS48165.1| chitinase-like protein-1, partial [Mytilus coruscus43]	446	25	355	376	74.22	27.13	1.00 E-42	153
CGI_10013462	540	3	216	gi|174428/239129|CB3_LOTGI RecName: Full= Chitin-binding_3 domains AltName: Full=Uncharacterized shell protein 14; Short=LUSP-14[Lgigantea3]	833	8	213	214	24.73	35.98	3.00 E-42	156
